# Local Geomorphological Gradients and Land Use Patterns Play Key Role on the Soil Bacterial Community Diversity and Dynamics in the Highly Endemic Indigenous Afrotemperate Coastal Scarp Forest Biome

**DOI:** 10.3389/fmicb.2021.592725

**Published:** 2021-02-24

**Authors:** Henry Joseph Oduor Ogola, Ramganesh Selvarajan, Memory Tekere

**Affiliations:** ^1^Department of Environmental Science, University of South Africa, Florida Science Campus, Roodepoort, South Africa; ^2^School of Agricultural and Food Sciences, Jaramogi Oginga Odinga University of Science and Technology, Bondo, Kenya

**Keywords:** coastal scarp forest, microbiome, bacteria, bulk soils, ecosystem dynamics, plant endemism

## Abstract

Southern Afrotemperate forests are small multi-layered and highly fragmented biodiversity rich biomes that support unique flora and fauna endemism. However, little is known about the microbial community and their contribution to these ecosystems. In this study, high throughput sequencing analysis was used to investigate the soil bacterial community structure and function, and understand the effect of local topography/geomorphological formations and land use patterns on a coastal scarp forest. Soil samples were collected from three forest topography sites: upper (steeper gradients, 30–55°; open canopy cover, <30%), mid (less steep, 15–30°; continuous forest canopy, >80%), and lower (flatter gradient, <15°; open canopy cover, 20–65%), and from the adjacent sugarcane farms. Results indicated that forest soils were dominated by members of phyla *Proteobacteria* (mainly members of *α-proteobacteria*), *Actinobacteria*, *Acidobacteria*, *Firmicutes*, and *Planctomycetes*, while *Actinobacteria* and to a lesser extent *β-proteobacteria* and *γ-proteobacteria* dominated SC soils. The core bacterial community clustered by habitat (forest vs. sugarcane farm) and differed significantly between the forest topography sites. The *Rhizobiales* (genera *Variibacter*, *Bradyrhizobium*, and unclassified *Rhizobiales*) and *Rhodospirallales* (unclassified *Rhodospirillum* DA111) were more abundant in forest mid and lower topographies. Steeper forest topography (forest_upper) characterized by the highly leached sandy/stony acidic soils, low in organic nutrients (C and N) and plant densities correlated to significant reduction of bacterial diversity and richness, associating significantly with members of order *Burkholderiales* (*Burkholderia-Paraburkholderia*, *Delftia*, and *Massilia*) as the key indicator taxa. In contrast, changes in the total nitrogen (TN), soil organic matter (SOM), and high acidity (low pH) significantly influenced bacterial community structure in sugarcane farm soils, with genus *Acidothermus* (*Frankiales*) and uncultured *Solirubrobacterales* YNFP111 were the most abundant indicator taxa. Availability of soil nutrients (TN and SOM) was the strongest driver of metabolic functions related to C fixation and metabolism, N and S cycling; these processes being significantly abundant in forest than sugarcane farm soils. Overall, these results revealed that the local topographical/geomorphological gradients and sugarcane farming affect both soil characteristics and forest vegetation (canopy coverage), that indirectly drives the structure and composition of bacterial communities in scarp forest soils.

## Introduction

The global significance of forests as carbon sinks, sanctuaries of biodiversity and providers of ecosystem services cannot be overestimated. In the last three decades, there has been increased interest in ecology of forest soils, partly due to the interlinkage of above-ground and below-ground soil microenvironments in contributing to the biodiversity-ecosystem functioning of forests. There is a general consensus that forest soils are a hotspot of dynamic bioprocesses facilitated by complex interactions of a multifaceted microbiome and above-ground vegetation critical in executing the key ecosystem functions such as primary production, litter decomposition, and organic matter mineralization (Sun et al., 2016; Lladó et al., 2017; Osburn et al., 2019; Song et al., 2020).

Soil microbiota are the main actors driving forest ecosystem functioning, inhabiting multiple habitats with specific properties such as bulk soil, rhizosphere, litter, and deadwood habitats, where their immense taxonomic abundance and functional diversity are greatly shaped by intrinsic soil abiotic and biotic properties, plant phenology, among other factors (Sabais et al., 2011; Lladó et al., 2018; Melo et al., 2019). Specifically, soil microbiomes play key role in the degradation of the recalcitrant organic matter in soils and plant litters, contributing to soil processes critical in the transfer of matter and energy through the decomposition of dead plant biomass, soil weathering and participating in beneficial plant roots-mycorrhizal interactions. Additionally, variation in soil microbial communities also have been reported to play important role in altering the environmentally driven selection pattern of plant functional traits critical to community succession such as root and leaf traits (Orwin et al., 2010; Bardgett et al., 2014; Chai et al., 2019). Previous studies in other forest ecosystems have reported that the soil microbial diversity is dependent on the local geomorphological factors (Feeser et al., 2018), soil geochemical properties (Lauber et al., 2009; Talbot et al., 2014; Sun et al., 2016; Lladó et al., 2017), vegetation composition (Sun et al., 2016; Chai et al., 2019; Osburn et al., 2019), season (Ali et al., 2019), and anthropogenic impacts (Frossard et al., 2017; Brinkmann et al., 2019; Osburn et al., 2019). The stochastic effect of these factors on the microbial community structure contributes to the variability of microbiomes within and among different forest habitats key to ecosystem functioning. Therefore, understanding the mechanisms shaping microbial community structure and functional attributes is essential for predicting soil functional capacity and ecosystem functions.

South Africa is endowed with unique forest ecosystems, extending from the subtropical moist broadleaf forest biome in Knysna-Tsitsikamma forest complex in Eastern and Western Cape provinces to inland Afromontane forest types of the KwaZulu-Natal, Mpumalanga, and Limpopo Province. These indigenous forests are very small in size and highly fragmented Afrotemperate biomes, covering less than 0.25% of the total land area (Berliner, 2009). In terms of known biodiversity, these forests are one of the most diverse terrestrial ecosystems with the highest biodiversity of any temperate forested region in the world (Silander, 2000), and are also home to numerous endemic species of flora and fauna (Matthews et al., 1993; Edwards et al., 2008; Clark et al., 2009, 2011; Lötter et al., 2014). Additionally, various studies have postulated that ecosystem functioning is positively related to plant diversity (Mucina et al., 2006; Neuschulz et al., 2011; Botzat et al., 2015; Grass et al., 2015). The coastal scarp forests (also known as Pondoland scarp forests), the focus of this study, generally associated with steep slopes, scarps, and cliffs of deeply incised gorges of the river dissecting the coastal plateau of the Wild Coast of the Eastern Cape Province and south western seaboards of KwaZulu-Natal, have been reported to be the most valuable indigenous forest type in South Africa due to their relict character and characteristic high diverse mixture of species, as well as scarp forest specific high plant and fauna endemics (Mucina et al., 2018). Mucina et al. (2018) reported seven-habitat level plant communities that could be grouped into three coastal scarp forest subtypes. It is hypothesized that the local geomorphology controls the soil formation processes, including small-scale patterns of soil texture and concentrations of mineral nutrients that drives plant diversity dynamics and endemism. Despite their high endemism of flora and fauna, coastal scarp forests are also under land-use intensification pressures similar to other forest ecosystems worldwide (Grass et al., 2015; Mangwale et al., 2017; Smithwick, 2019). These ranges from the growing human needs in rural tribal areas, high intensity farming interests, and uncontrolled exploitation, grazing and burning of forests on farms, that has greatly influenced forest coverage. For example, large percentage (>85%) of smooth landscape of scarp forest ecosystem in KwaZulu Natal Province, South Africa, are under large scale sugarcane farming, with the forest occurring only in the river gorges.

Overall, the scarp forests are dominated with soils ranging from deeper soils with a higher content of clay (and silt), C, K, P, Na, Ca, and Mg in smoother landscape, to highly acidic, stony and well-leached soils, low in nutrient reserves and cation exchange capacity but with a notable accumulation of litter (Mucina et al., 2018). Collectively, these factors coupled with climatic factors may account for the ecosystem productivity and drives the high plant diversities and scarp forest specific endemism. It can be hypothesized that soil microbial community intrinsic to indigenous scarp forest are also a major component of these ecosystem biodiversity and primary drivers of biogeochemical processes such as biomass decomposition, contributing significantly to biodiversity-ecosystem functioning and the observed above-ground vegetation biodiversity endemism. However, to our knowledge, little information is available on the contribution, and the interrelationship between below-ground soil ecology, microbial diversity, and above-ground plant biodiversity in scarp forest biomes. Further, loss in microbial diversity in forest scarp forest biomes as consequence of increasing anthropogenic activities and climate change would likely alter the capacity of microbes to sustain multiple above‐ and below-ground ecosystem functions (Bodelier, 2011; Classen et al., 2015).

To gain a more comprehensive understanding of the scarp forest ecology and functional potentials of bacterial communities, a suite of analyses on independent forest floor soil samples in a “pristine” natural Scarp forest in Oribi Gorge Nature Reserve was performed. Overall, the study aimed at unraveling the bacterial community ecology of scarp forest floor bulk soils along the typical topographical/geomorphological gradients (in terms of slope, elevation, and plant canopy coverage). Specifically, we hypothesized: (1) a decrease in bacterial taxonomic abundance at high and steep elevation following classic views on biodiversity gradients (Rahbek, 1995); (2) changes in the forest soil bacterial communities’ structures according to variations of ecological niches along the local topographical/geomorphological gradients, and sites bordering sugarcane farm (SC) impacted by land use intensification activities.

## Materials and Methods

### Study Area and Sampling

**Figure 1:**
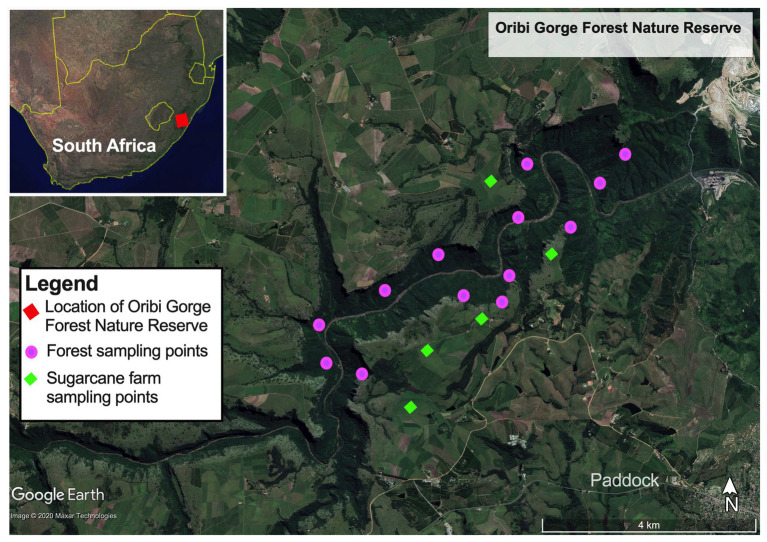
Map of Oribi Gorge Nature Reserve coastal scarp forest showing its location in the eastern seaboard of South Africa (inset **upper right panel**) and the sampling points of forest soils and sugarcane farms bordering the forest.

Samples for the study were collected from Ezemvelo Parks protected Oribi Gorge Natural Reserve (30° 41'–30° 45’S, 30° 10'–30° 18'E), one of the small and fragmented paleoendemic natural scarp forests in the Natal – Pondoland coastal region (Figure 1). This forest is located about 21 km from Port Shepstone in KwaZulu Natal Province, South Africa. The area has an annual temperature range between 13 and 23°C (Makinde and Abiodun, 2020), mean annual rainfall of 1,120 mm, with wetter season occurring in October to March. The forest covers approximately 1,600 ha, occurring on gently sloping to very steep mid-slopes (1–37°) at altitudes of 205–415 m of deeply incised gorges and canyons drained by Umzinkulwana River, surrounded by large sugar cane plantations and grasslands. Phytogeographically, the forest is dominated with thorny elm [*Chaetacme aristate* Planch.], *Prosphytochloa prehensilis* (Nees) Schweick, African Oak [*Drypetes arguta* (Müll.Arg.) Hutch.], Silver-leaved Milkplum [*Englerophytum natalense* (Sond.) T.D.Penn.], Dwaba-berry [*Monanthotaxis caffra* (Sond.) Verdc.], and Bastard White Ironwood (*Drypetes gerrardii* Hutch.), as the common plant communities (Mucina et al., 2018). The forest is also characterized by up to 80% continuous canopy cover, but mainly having a more open upper (20–65% canopy) and lower (15–50% canopy) shrub layers. The main species in the sparse herb layer include *P. prehensilis* (Nees) Schweick, Green cliff brake or Common lip fern [*Cheilanthes viridis* (Forssk.)], and King fern [*Todea barbara* (L.) T.Moore]. On average, forest floor is covered with 50–70% litter material, consisting up to 17% dead organic wood material.

Soil samples were collected in January 2018 from three different locations representing the typical topographical formations in terms of mean elevation and gradient (slope degree, aspect, and position) and canopy coverage in the forest. Among them, UL (forest upper level) had an elevation between 290 and 415 m asl, with steep gradient (30–55°), open canopy cover (<30%) often occurring between the rocky outcrops of the gorge. ML (forest mid-level) had elevations between 230 and 290 m asl, a moderate gradient (10–30°) and more robust vegetation cover characterized by a closed continuous canopy cover (up to 80%). ML also had a forest floor having well-drained soil covered with forest litter (50–70%) and dead wood material (17%). On the other hand, LL (forest lower-level) had an elevation of 205–230 m asl having similar characteristic of ML on the upper edges but having sparse and open canopy coverage (20–65%) on the lower reaches bordering the riverbed at the bottom the gorge. The lower reaches of ML had highly leached sandy soils, low in forest litter and devoid of dead woody material. In addition to forest, soil samples were collected from the SC bordering the forest as control to evaluate the impact of land intensification on the forest soil quality. These sugarcane farms are located on the upper smooth landscape sandwiching and draining into the forested gorge.

In each site, soil samples were collected in at least four quadrats (5 × 5 m) along a 100 m linear transect within the forest, that reflected the accessible four typical topographical formations of the forest (Figure 1). At the four corners and the center of each quadrat, soil samples (~150–200 g) were collected randomly from the 0 to 20 cm depth using a soil corer and mixed to form one replicate. When collecting the soil sample where a tree stump or rock occurred at the sampling point, a slightly off-center sampling point was selected. In total, samples collected included: four composite replicates each for forest_mid (ML) and forest_upper (UL); and five composite replicates each for forest_lower (LL) and sugarcane_farm (SC). Collected soil samples were transported on ice (<4°C) to the laboratory within 12 h of collection, for further analysis.

### Soil Geochemical Parameters

In the laboratory, non-soil materials (gravel, larger plant and woody materials) were removed manually from each sample and the sample was divided into two portions. Samples for DNA metagenomic analysis were prepared by transferring aseptically 20 g of well-mixed soil from each of the five cores in a quadrat to get a composite sample for each site, into sterile plastic bags. These samples were stored at −80°C until analysis. The other soil subset was air-dried, milled and sieved through a 2-mm mesh for soil geochemical analysis. Soil geochemical factors, including pH, electrical conductivity (EC), water content, total carbon (TC), soil organic matter (SOM), total nitrogen (TN), and metals (Al, Ca, Fe, K, Mg, Na, and Zn), were quantitatively measured as previously described by Sekhohola-Dlamini et al. (2020).

### DNA Extraction and Purification

Total DNA was extracted from all samples using the Fecal/Soil Total DNA™ extraction kit (Zymo Research Corporation, CA, United States). Briefly, about 2 g of each of the collected samples was initially mixed with 5 ml phosphate buffered saline (PBS, pH 7.4). The mixture was agitated by vortexing and allowed to stand for 15 min at room temperature to dislodge bacterial cells adhering to solid soil particles. An aliquot of 400 μl of resultant supernatant was then used for extraction according to manufacturer’s instructions and stored at −20°C prior to further analysis. For each soil sample, DNA was extracted in triplicates and pooled together before use for downstream analysis. The quantity and quality of extracted DNA was checked on Biodrop μLite spectrophotometer (Biochrom Ltd., Cambridge, United Kingdom) and agarose gel electrophoresis, respectively. Extracted DNA used for downstream PCR and sequencing analysis had A_260_:A_280_ ratios between 1.8 and 2.0 and DNA concentrations of 20–150 ng/μl.

Initial PCR amplification of the whole variable region of bacterial 16S rRNA was done using the universal primers 27F (5'-AGAGTTTGATCCTGGCTCAG-3') and 1429R (5'-TACGGYTACCTTGTTACGACTT-3'; Frank et al., 2008). Each 25 μl reaction volume contained 0.5 μM of each primer, 1X OneTaq® Hot Start Master Mix (New England Biolabs, Ipswich, MA, United States) and 20 ng DNA. PCR was performed under following cycling conditions [95°C, 5 min; 32 × (95°C, 1 min; 55°C, 1 min; 72°C, 1 min); 72°C, 7 min; 4°C, ∞], and resultant amplicons checked on a 1.5% agarose gel. A second PCR amplification to cover the bacterial 16S rRNA V1-V3 hypervariable region was performed using 27F and 518R primer pairs, fused with MiSeq adapters and heterogeneity spacers compatible with Illumina indexes for multiplex sequencing, as described by Fadrosh et al. (2014).

### Bacterial 16S rRNA Gene Sequencing and Processing

Cleaning of the resultant PCR product, index library preparation, pooling and sequencing on Illumina Miseq 250® to generate paired 300-bp high-quality reads of the V1-V3 region were performed as described by Selvarajan et al. (2019). Briefly, amplified PCR product was initially cleaned using AMPure XP magnetic beads (Beckman Coulter, Massachusetts, United States) according to manufacturer’s instructions. After purification, Illumina sequencing dual-index barcodes were added to the amplicon targets using full complement of Nextera XT indices (Illumina Inc., San Diego, CA, United States) in 25-μl PCR reaction [95°C, 3 min; 8 × (95°C, 30 s; 55°C, 30 s; 72°C, 30 s); 72°C, 5 min; 4°C, ∞]. PCR amplicons were cleaned using AMPure XP beads, and the fragments size (~630 bp) was validated using Bioanalyzer DNA 1000 chip (Agilent, Santa Clara, CA, United States) and quantified using Qubit™ dsDNA HS Assay kit (Thermo Fisher Scientific, Waltham, MA, United States). About 5 μl aliquot of purified equimolar DNA (4 nM) from each library were pooled into a single amplicon library and stored at −20°C until sequencing. Pooled libraries were subsequently sequenced on Illumina MiSeq 250 platform with v3 chemistry (2 × 300 cycles kit; Illumina Inc., San Diego, CA, United States). The raw high throughput sequencing data was deposited into the NCBI Sequence Read Archive database BioProject ID PRJNA650217.

### Statistical Analyses

Processing of the trimmed raw sequences (fastq files) was performed using next-generation sequencing short reads (*ngsShoRT*) trimmer algorithm (Chen et al., 2014) before being merged and analyzed on Mothur pipeline v.1.40.0 (Schloss et al., 2009), as described by Selvarajan et al. (2019). During the analysis, sequence reads were quality filtered, chimeric sequences removed using UCHIME algorithm (Edgar et al., 2011), classifying quality reads using the Naïve Bayesian classifier algorithm (Wang et al., 2007) against the SILVA database version 132 (Quast et al., 2013). Pairwise distance (Euclidean distance) matrix algorithm based on mothur’s “*dist.seqs*” command with parameter “*cutoff = 0.03*” and the “*cluster*” command using the default “*furthest neighbor*” option were used to cluster and assign operational taxonomic units (OTUs) at phylum, class, order, family, and genus levels (Schloss et al., 2009). OTUs unassigned or assigned to mitochondria and chloroplast were removed. Only singletons contributing at least 0.01% of total sequence abundance in any sample were deemed detected and retained for further statistical analysis and data interpretation, and these were further divided into minor (<0.1%) and major (≥0.1%) for ease of description.

The major OTUs at different taxonomic levels (phylum, class, order, family, and genus) were used to generate stacked bar charts and heatmap using *ggplot2* (Wickham, 2016) and *heatmap.2* packages (Warnes et al., 2019) in R version 3.6.1 (R Core Team, 2019), respectively, to visualize the variations and distributions of bacterial communities. Alpha diversity indices were calculated at the genetic distance of 0.03 using the plot_richness function of *phyloseq* (McMurdie and Holmes, 2013). The distances were then plotted in *ggplot2* (Wickham, 2016), and variations tested statistically using ANOVA, *post hoc* tests (Tukey-Kramer at 0.95), an effect size (Eta-squared) and multiple test correction using Benjamini-Hochberg false discovery rate (FDR) procedure. β-diversity based Bray-Curtis dissimilarity distance, hierarchical clustering and principal coordinates analysis between the sampling sites was performed using *vegan* package on R (v3.6.1; R Core Team, 2019). Non-metric multidimensional scaling (NMDS) analysis was also conducted to explore the bacterial community composition based at both the phylum and OTU levels at Bray-Curtis dissimilarity between four sample sets. The direction and length of the vectors of taxa and soil geochemical factors (pH, EC, TC, TN, SOM, BD, TP, Al, Ca, Fe, K, Mg, Na, and Zn) were computed by Bray-Curtis distances using the “envfit()” function in the *vegan* package. The significance of the convergence between forest topography types and sugarcane farm soil bacterial community was validated by performing nonparametric analysis of similarity (ANOSIM) and adonis PERMANOVA (Anderson and Walsh, 2013) using *vegan* package in R. While ANOSIM generates an *R* statistic and *p* value, adonis PERMANOVA gives both *F* statistic, *R*^2^ and *p* values. Both *R* and *R*^2^ are statistics for compositional dissimilarity, where higher values indicate dissimilarity.

The “*indicspecies*” package in R was used to identify bacterial OTU, which are significantly associated with different forest topography and sugarcane farm (Cáceres and Legendre, 2009). The *point biserial correlation coefficient* was calculated for all identified genera and all taxa with significant (*p* ≤ 0.05) associations were visualized in the network generated using the *edge-weighted spring-embedded layout algorithm* in Cytoscape 3.5.1 (Shannon et al., 2003) as described by Brinkmann et al. (2019). Finally, PICRUSt2 (Phylogenetic Investigation of Communities by Reconstruction of Unobserved States) software package (Langille et al., 2013) was used to predict and understand the potential functional capabilities and difference between the forest soil bacterial communities. Toward this, the nearest sequenced taxon index (NSTI) value was used to validate the reliability of predicted functional and metabolic pathways. The heatmap of the predicted relative abundances of genes related to different functions was generated using *heatmap.2* package in R (v3.6.1; R Core Team, 2019).

## Results

### Soil Physicochemical Factors Variability

The forest topography type soils, especially UL exhibited significantly unique soil properties such as pH, bulk density (BD), TC, SOM, total phosphorous (TP), and mineral content (Al, K, Fe, and Mg) than mid (ML) and lower forest (LL) levels (Table 1). The soil pH varied from 4.82 to 5.44, with UL having significant (*p* < 0.05) lower pH than other forest topography samples. Similarly, the forest_upper had sandy soils with significantly (*p* < 0.05) higher BD and metal contents (Al, Ca, Fe, K, and Mg). Overall, the less steep sites (forest_lower and mid) had deeper soils (BD < 1.0 g/cm^3^) with significant higher organic nutrient levels (TC, C/N, SOM, and TP) compared to the steeper forest upper soils. In contrast, no significant (*p* > 0.05) difference were observed for soil moisture (SM) and TN. Comparatively, the sugarcane farm soils were significantly (*p* < 0.05) more acidic in nature (pH < 4.0), having higher levels of TN and Ca but low in TC, SOM, and TP, than forest soils (Table 1). Nevertheless, it had comparable levels of SM and metal content (Al, Ca, Fe, K, and Mg) to forest soil samples.

**Table 1:** Physicochemical properties of soils under different forest topography types.

Parameter	Forest_lower (LL)	Forest_mid (ML)	Forest_upper (UL)	Sugarcane farm (SC)
pH	5.32 ± 0.04*c*	5.44 ± 0.23*c*	4.82 ± 0.17*b*	3.73 ± 0.18*a*
SM (%)	10.7 ± 0.86*b*	10.9 ± 2.78*b*	11.7 ± 2.42*b*	11.6 ± 2.60*a*
BD (g/cm^3^)	0.87 ± 0.09*c*	0.92 ± 0.05*c*	1.73 ± 0.09*a*	1.26 ± 0.01*b*
TN (%)	0.47 ± 0.17*b*	0.44 ± 0.14*b*	0.42 ± 0.08*b*	1.50 ± 0.22*a*
TC (%)	5.51 ± 0.66*a*	7.47 ± 0.86*a*	2.79 ± 0.53*b*	0.95 ± 0.46*b*
C/N	14.3 ± 6.45*a*	15.0 ± 0.37*a*	6.76 ± 1.51*b*	0.63 ± 0.27*c*
SOM (%)	7.05 ± 1.13*b*	9.56 ± 1.49*a*	4.76 ± 0.92*c*	1.63 ± 0.80*d*
Ca (mg/kg)	0.47 ± 0.29*c*	3.21 ± 1.11*b*	5.94 ± 0.89*b*	14.3 ± 3.06a
Al (mg/kg)	87.4 ± 46.8*b*	94.7 ± 61.5*b*	287 ± 127*a*	75.7 ± 25.0*b*
Fe (mg/kg)	43.5 ± 31.5*b*	86.7 ± 28.9*b*	182 ± 90*a*	43.5 ± 6.03*b*
K (mg/kg)	4.65 ± 0.17*b*	12.4 ± 10.9*b*	33.4 ± 15.0*c*	9.08 ± 4.07*b*
Mg (mg/kg)	2.95 ± 2.37*c*	4.75 ± 2.90*b*	8.56 ± 3.12*a*	2.95 ± 0.41*c*
TP (mg/kg)	40.6 ± 5.87*a*	42.4 ± 3.67*a*	36.4 ± 2.38*b*	26.7 ± 5.13*c*

SM, soil moisture; BD, soil bulk density; TN, total nitrogen; TC, total carbon; C/N, the ratio of total organic carbon to total nitrogen; SOM, soil organic matter; and TP, total phosphorus. Different lowercase letters showed significant differences under different forest topography types (*p* > 0.05).

### Distribution of Taxa and Phylotypes

**Figure 2:**
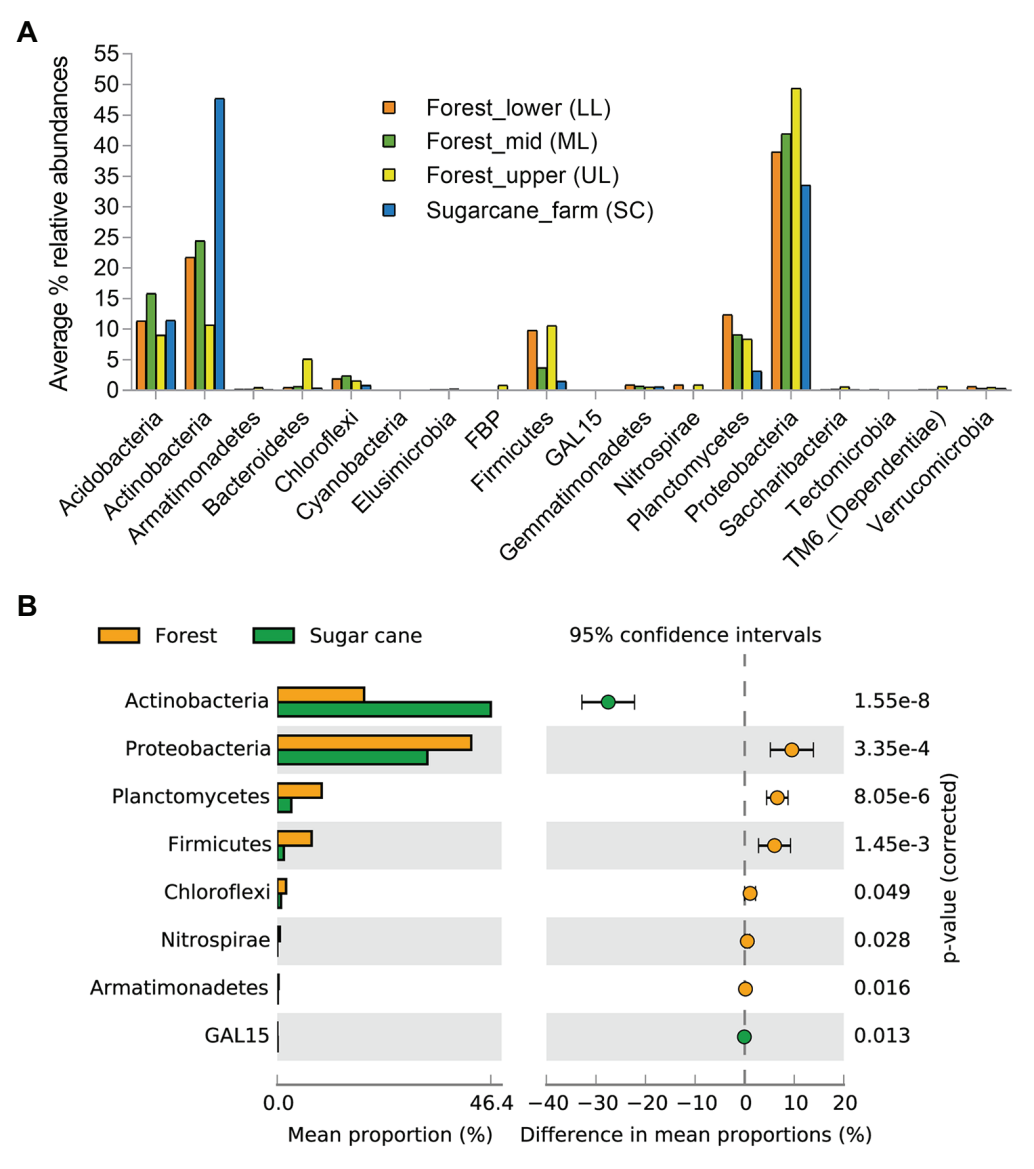
Relative abundances of major phyla level in the forest topography types and adjacent sugar cane farms **(A)** and **(B)** microbial phyla that exhibited significant differences (*p* < 0.05) between forest and sugarcane soils. Significance was determined by Fisher’s exact test with Storey’s false discovery rate (FDR) correction for multiple comparisons. Only taxa with difference between proportions >0.2 (i.e., considered large effect) are shown.

To characterize the bacterial community structure, DNA was isolated from 18 soil samples derived from all analyzed forest landscapes and an adjacent sugarcane farm. After removal of low-quality sequences and singletons, amplicon-based analysis of the V1-V3 of the bacterial 16S rRNA resulted in 385,006 high-quality sequences, ranging between 16,679 and 27,214 per sample with an average read length of 527 bp. Good’s coverage across the samples was >99%, indicating that the sampling depth was sufficient to estimate the microbial diversity enclosing all major bacterial group inhabiting the studied forest and land systems (Table 2). This was further supported by the rarefaction curves and rank abundance plots (Supplementary Figure 1) that asymptotically approached a plateau, suggesting that the curves accurately reflected the bacterial communities. The total bacterial domain consisted of 34 phyla, 108 classes, 181 orders, 363 families, and 776 genera. The dominant phyla included *Proteobacteria* (28.3–53.7%), *Actinobacteria* (6.2–51.2%), *Firmicutes* (0.55–19.88%), *Planctomycetes* (1.75–13.12%), *Acidobacteria* (7.14–19.46%), including *Chloroflexi* (0.37–7.89%), *Bacteriodetes* (0.05–16.04%), *Gemmatimonadetes* (0.21–1.68%), *Nitrospirae* (0–2.07%), *Verrucomicrobia* (0.15–1.37%), *TM6_Dependentiae* (0.01–1.97%), and *Saccharibacteria* (0.02–1.72%), with these group accounting for >99% of sequences from each forest topography types and sugarcane farm soil (Figure 2A). Overall, the bacterial composition at phylum level varied in relationship to the habitat type (forest and sugar cane). A significant enrichment (*p* < 0.05) of the phyla *Proteobacteria*, *Planctomycetes*, *Firmicutes* and to lesser extent *Chloroflexi*, *Nitrospirae*, and *Armatimonadetes* was found in the forest soil in contrast to increase in *Actinobacteria* and *GAL15* in the sugarcane soils (Figure 2B).

**Table 2:** Summary of OTU richness diversity indices of bacterial taxa in different forest landscape and adjacent sugar cane plantations^†^.

Habitat	Elevation code	Valid reads	Observed OTUs	Chao1	Simpson evenness	Simpson diversity (1/D)	Goods Coverage (%)
Forest soils	UL	23,183 ± 3,813	228 ± 21*a*	243 ± 27*a*	0.964 ± 0.001*ab*	27.9 ± 0.9*a*	99.1
	LL	19,552 ± 3,244	297 ± 37*b*	310 ± 36*b*	0.974 ± 0.005*a*	39.4 ± 7.6*b*	99.7
	ML	19,818 ± 3,441	338 ± 76*b*	347 ± 77*b*	0.975 ± 0.008*a*	44.3 ± 15.2*b*	99.8
Sugarcane farm soil	SC	22,881 ± 3,842	248 ± 41*ab*	264 ± 47*a*	0.959 ± 0.007*b*	24.9 ± 3.8*a*	99.4
*p*-value		0.310	0.019	0.037	0.004	0.011	0.465

† Sample comparison for diversity indices (OTUs, Chao1, Simpson evenness and diversity) were performed using rarefied datasets of 16,708 sequences representing the lowest number of reads in a sample. One-factor ANOVA was carried out to compare the sampling sites and *post hoc* (Tukey’s) tests applied. Different superscript letters within a column indicate significant differences at *p* ≤ 0.05.

### Bacterial Diversity and Differences in Community Structure Among the Forest Topographical Types

For statistical comparisons, the dataset was subsampled and rarefied to an even depth of 16,708 sequences (minimal number of sequences recorded). Overall, the Chao 1 estimated richness of the entire dataset was 1,111 unique OTUs (277 + 60 per sample; Table 2). However, all the calculated alpha diversity indices (observed OTUs, Chao1 estimator, Simpson evenness, and Inverse Simpson) were significantly different between sample types (ANOVA test; *p* < 0.05; Table 2). *Post hoc* Tukey tests revealed that the alpha diversity, with exception of the Simpson evenness, for forest_mid (ML) and forest_lower (LL) were consistently greater (Tukey’s, *p* < 0.05; Table 2) than for the forest_upper (UL), indicating that forest topography plays an important role in determining soil bacterial richness and diversity. Overall, the bacterial richness and diversity of sugarcane farm soil was also significantly different (Tukey’s, *p* > 0.05) from forest soil.

The relative abundance of each bacterial taxonomic group varied among the forest topographical types. At class level, *α-proteobacteria*, *Actinobacteria*, *Planctomycetacia*, *Acidobacteria*, *β-proteobacteria*, *γ-proteobacteria*, *Bacilli*, *Acidimicrobiia*, *δ-proteobacteria*, and *Sphingobacteriia* were dominant in all forest samples. However, subtle variation of the taxa among the forest topography sites and sugarcane farm were discernible (Supplementary Figure 2). Both forest_lower and forest_mid exhibited similar bacteria taxonomic profile, with higher abundance of *α-proteobacteria* (26.7 and 27.9%, respectively), *Thermoleophila* (15.7 and 17.6%), *Acidobacteria* (8.7 and 9.4%) *Actinobacteria* (15.5 and 10.1%), and *Acidimicrobiia* (4.1 and 4.0%). Conversely, the *γ-proteobacteria* and *Bacilli* phylotypes were abundant in forest_upper (8.6 and 7.4%) and sugarcane farm (8.9 and 7.8%) but less abundant in the forest_lower (3.8 and 2.7%) and forest_mid (3.7 and 4.7%). Additionally, sugarcane farm exhibited ~3–10-fold higher abundance of *β-proteobacteria* (13.7%) and *Sphingobacteriia* (3.7%) than all forest samples, whereas forest_upper reported highest enrichment of *Planctomycetacia* (11.1%; Supplementary Figure 2).

### Impact of Topography-Associated Soil Characteristics on Bacterial Diversity and Community Composition

**Figure 3:**
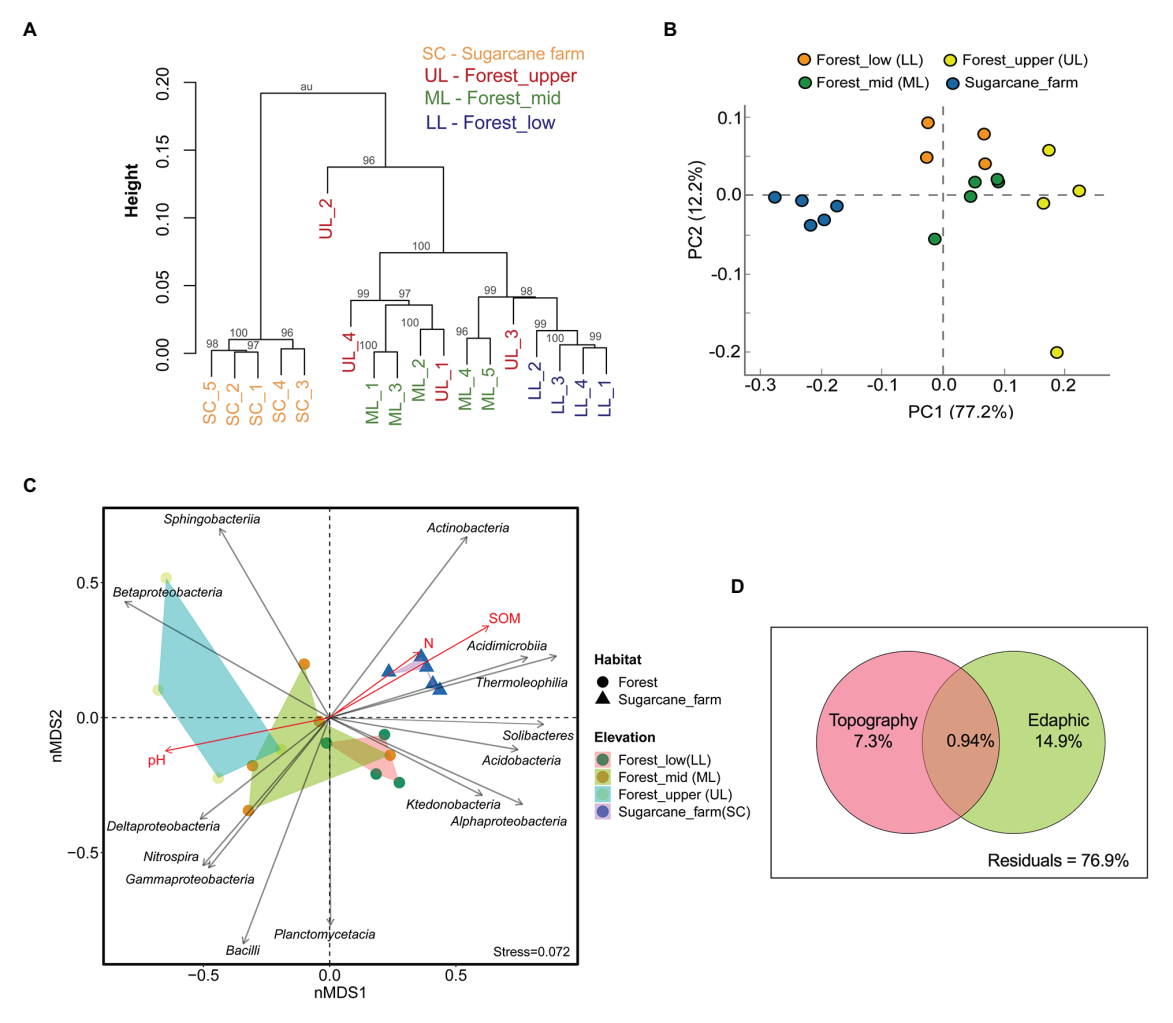
Beta diversity analysis of bacterial communities under three forest topography types and sugarcane farm. **(A)** Hierarchical clustering based on operational taxonomic unit (OTU) abundance-based Bray-Curtis similarity coefficients. **(B)** Principal Component Analysis (PCA) based taxonomic profile at phylum level. **(C)** Non-metric multidimensional scaling (NMDS) analysis showing relationships among forest and sugar cane farm bacterial community composition at the class level with topography and environment variables. Stress value including ANOSIM test *R* and *p*-values are shown at the bottom-left of the plot. **(D)** Venn diagram representing variation partitioning of bacterial communities explained by forest topography type and edaphic variables.

Hierarchical clustering (Figure 3A) and Principal Component Analysis (PCA) ordination plot (Figure 3B) based on taxonomically similar microbial profiles showed grouping into specific forest topography and sugarcane bulk soil clusters, implying distinct microbial community profiles. In the PCA analysis, PC 1 explained 77.2% and PC 2 explained 12.2% of the total bacterial variations (Figure 3B). However, there was no clear separation between forest_lower and forest_upper samples. To further determine the statistical significance of the observed spatial heterogeneity, nMDS (based on Bray-Curtis dissimilarity) and PERMANOVA analysis of community abundance at class and genus level were performed (Figure 3C; Supplementary Table 1). The results of statistical analysis of beta diversity were in agreement with those from the ordination analysis and the hierarchical cluster analysis. The NMDS plots revealed that the core community at class level clustered by habitat (i.e., forest vs. sugarcane farm; ANOSIM, *R* = 0.643, *p* < 0.001, PERMANOVA, *F* = 21.42, *p* = 0.0003) and topography (ANOSIM, *R* = 0.632, *p* < 0.004; PERMANOVA, *F* = 6.49, *p* = 0.0001; Supplementary Table 1). Similarly, clustering of bacterial genera by habitat and elevation was observed, however, the separation was not typically large (ANOSIM: *R* < 0.50, *p* < 0.05; Supplementary Table 1). Stress of the nMDS ordination was 0.17, a value that reveals the degree of correspondence between the data points, where a stress value of zero indicates perfect fit and a value close to one is a pointless relationship. Pairwise Spearman rank correlation of bacterial diversity at class level also showed that forest_mid was significantly different from forest_lower (ANOSIM, *R* = 0.289, *p* = 0.003) and forest_upper (ANOSIM, *R* = 0.489, *p* = 0.028), result also supported by adonis PERMANOVA analysis. Similarly, the sugarcane farm exhibited significant and high dissimilarity (*R* > 0.8, *p* < 0.01) from all the forest topography types (Supplementary Table 1).

Core microbiome analysis demonstrated that unique OTUs differed within the three topography types and between forest and sugarcane farm (Supplementary Figure 3). Within the forest sites, the number of site-specific OTUs ranged from 5 (forest_upper) to 12 (forest_mid), and 23 OTUs (46.6% of total abundance) were shared among all forest sites. The shared OTUs were composed of a number of bacterial groups, including *Acidobacteria* (two OTUs; assigned to Subgroup 1 and 2), *Proteobacteria* (12 OTUs; assigned to genera *Bradyrhizobium*, *Variibacter*, *Achromobacter*, DA111, *Pseudomonas*, and unclassified *Rhizobiales*), *Actinobacteria* (one OTU; an unclassified *Solirubrobacteriales*), *Firmicutes* (three OTUs; assigned to genus *Paenibacillus* and unclassified *Bacillales*), *Planctomycetes* (three OTUs; assigned to genus *Singulispaera*, *Gemmata*, and unclassified *Planctomyceteae*), and *Armatimonadetes* (one OTU; an unclassified *Armatimonadales*). On the other hand, forest and sugarcane farm shared 42 OTUs, with sugarcane and forest having 22 and 9 unique OTUs, respectively. The major unique OTUs in sugarcane farm soils were mainly members of phylum *Acidobacteria* (genera *Candidatus_koribacter*, *Edaphobacter*, and *Solibacteraceae*), *Chloroflexi* (*Ktedobacterales*), and *Actinobacteria* (*Solirubrobacter*, *Bryobacter*, *Actinospica*, *Corynebacteriales*, and *Jatrophihabitans*).

**Figure 4:**
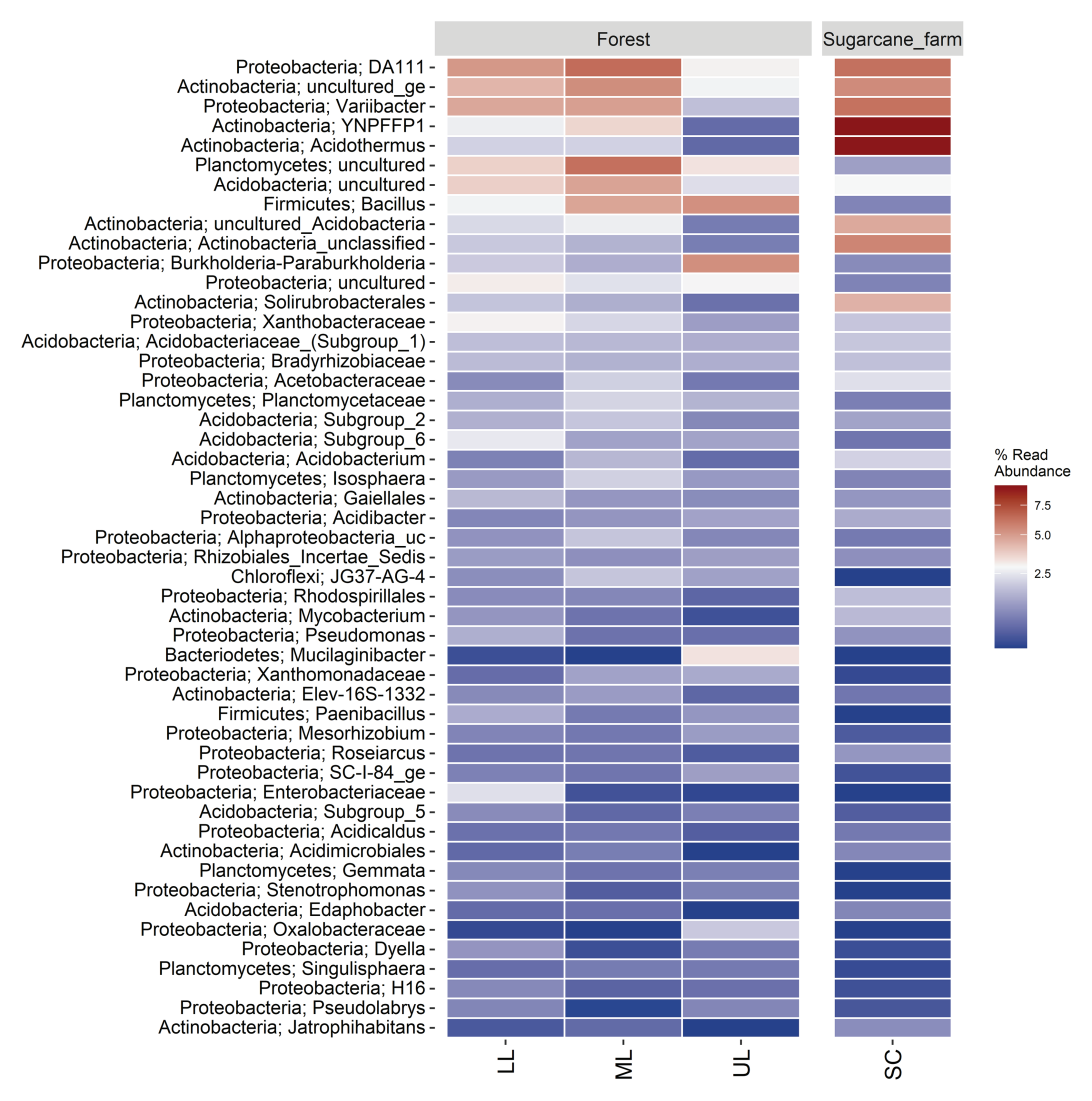
Percent relative abundance of top 50 genera in the forest topography sites and sugarcane farm (SC). LL, forest_lower; ML, forest_mid; UL, forest_upper; and SC, sugarcane farm. Heatmap was generated by ampvis2 package (Andersen et al., 2018) in R computing environment version 3.5.2.

A heatmap analysis of the top 50 genera was also used to investigate the differences in the soil bacterial communities of the forest topography types (Figure 4). Samples of forest_lower (LL) and forest_mid had *DA111*, uncultured *Actinobacteria*, and *Varibacter* as the highly enriched genera, whereas forest_upper had *Bacillus* and *Burkholderia-Paraburkholderia* as the dominant OTUs. The abundance of the OTUs also varied between forest and sugarcane farm. For example, the sugarcane farm site was dominated by *Acidothermus* and YNPFFP1, followed by lower abundance of uncultured *Plantomycetes*, *Acidobacteria*, *Bacillus*, and *Burkholderia-Paraburkholderia* than forest samples.

The analyis of vector-fitting of environmental variables to the NMDS space indicated that several environmental variables correlated with the ordination space defined by the bacterial class composition (Figure 3C). The major physicochemical characteristics driving soil bacterial community composition in different forest topography types, especially for forest_mid and forest_upper, was pH (*R*^2^ = 0.6703, *p* = 0.001). In contrast, both soil organic matter SOM (*R*^2^ = 0.7311, *p* = 0.007) and soil total N (*R*^2^ = 0.1130, *p* = 0.039) affected significantly soil bacterial diversity in sugarcane farm soils (Supplementary Table 2). To assess the relative importance of the spatial (forest topography type) and environmental (edaphic factors) variables on community composition, variation partitioning and RDA analysis using Monte Carlo permutation tests (999 unrestricted permutations) followed by forward selection to remove the non-significant variables from each of the explanatory sets was performed. Results revealed that in forest soil samples, edaphic variables (i.e., pH, SOM, and N) and forest topography type explained 22.2% of the total variation (*p* < 0.05) of bacterial communities, of which largest proportion (14.9%) of total variation (*p* < 0.05) was explained by soil variables alone (Figure 3D). Around, 7.3% of the bacterial community variation in the forest soil could be explained by forest topography type.

### Indicator Taxa for Specific Forest Topography Types

**Figure 5:**
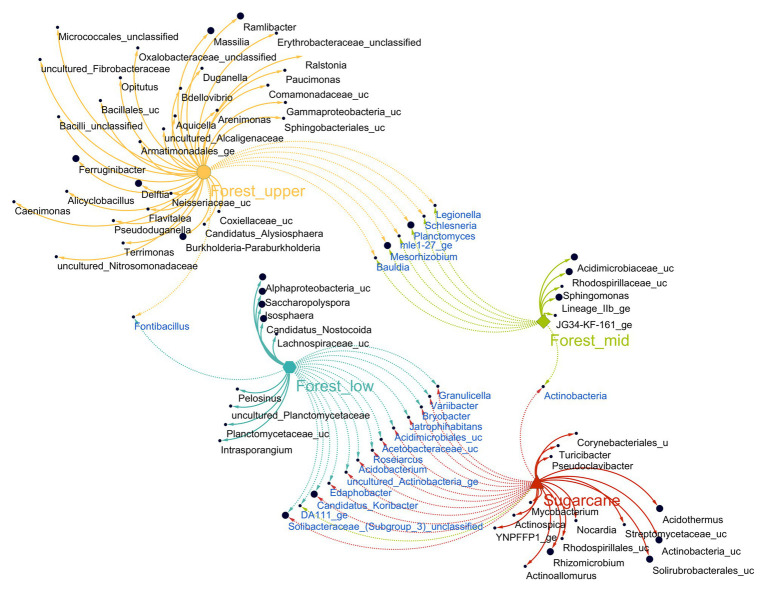
Association network of significantly abundant soil fungi in different land use systems [multipatt function in indicspecies package in R (Cáceres and Legendre, 2009)]. Node sizes represent the average relative abundance of OTUs in the data sets. Edges represent the association patterns of individual OTUs with the land use systems, and their lengths show the weight of the associations (edge-weighted, spring-embedded layout). The association strength of significant genera is indicated by different edge lengths varying between 0.09 and 0.79.

To identify OTUs sensitive to specific forest topography types and sugarcane site, indicator species analysis based on the point biserial correlation was performed. The method identified 61 OTUs had significant associations (point biserial correlation coefficient *R* > 0.4, *p* < 0.05) with a particular forest topography type or its combinations (Figure 5; Supplementary Table 3). These indicator OTUs accounted for 10.3% of the total numbers of OTUs, with forest_upper having most indicator OTUs (38) with relative abundance of 17.3%, followed by forest_lower (nine OTUs, with relative abundance of 7.2%) and forest_mid (seven OTUs, with relative abundance of 0.1%). Majority of indicator taxa of forest_upper soils (constituting of 11.4% of the total number of sequences) comprised OTUs belonging to class *β-proteobacteria*, specifically order *Burkholderiales* (*Burkholderia-Paraburkholderia*, *Delftia*, *Pseudoduganella*, *Massilia*, *Ramlibacter*, *Caenimonas*, unclassified *Oxalobacteraceae*, *Ralstonia*, unclassified *Alcaligenaceae*, and *Paucimonas*). Other indicator taxa belonged to class *γ-proteobacteria* (*Arenimonas*, *Aquicella*, and unclassified *Armatimonadales*), *α-proteobacteria* (*Candidatus Alysiosphaera* and *MNG7*), *Verrumicrobia* (*Opitutus*), *Bacilli* (*Alicyclobacillus* and *unclassified Bacillales*). and phylum *Bacteriodetes* (*Terrimonas*, *Ferruginibacter*, *Flavitalea*, and unclassified *Sphingobacteriales*). The forest_mid soils contained OTUs belonging to phylum *Actinobacteria* (*Saccharopolyspora* and *Candidatus-Nostocoida*), *Planctomycetes* (*Isosphaera* and uncultured *Planctomyceteae*), *Acidobacteria* (Subgroup 13), and *Firmicutes* (*Pelosinus* and unclassified *Lachnospiraceae*), while members of genera *Intrasporangium*, *Sphingomonas*, unclassified Subgroup 6, family *Acidomicrobiaceae* and *Rhodospirillaceae* were the main indicator OTUs in the forest_lower soils.

In sugarcane farm soils, 18 distinct indicator OTUs (accounting for 37.2% of the total number of sequences) were identified (Figure 5; Supplementary Table 3). These included taxa belonging to phylum *Actinobacteria* (12 OTUs, with a relative abundance of 31.9%), *Firmicutes* (two OTUs), *Proteobacteria*, mainly class *a-proteobacteria* (two OTUs), *Deinococcus_Thermus* (one OTU), and *Bacteria incertae sedis* (one OTU). Major actinobacterial indicator taxa were members of order *Solirubrobacteriales* (uncultured *Solirubrobacteriales* YNPFFP1 and unclassified *Solirubrobacteriales*) and *Frankiales* (*Acidothermus*), whereas the genus *Rhizomicrobium* and unclassified *Rhodospirillales* were the only proteobacterial indicator taxa identified (Supplementary Table 3). Additionally, significant associations (point biserial correlation coefficient *R* > 0.4, *p* < 0.05) of eight taxa detected between different forest topography site cross combinations were observed. In contrast, significant associations of 14 taxa between forest and sugarcane farm soil cross combination was observed, with bulk of the shared taxa (13) detected between forest_lower and sugarcane farm samples only (Figure 5).

### Prediction of Bacterial Community Functions

**Figure 6:**
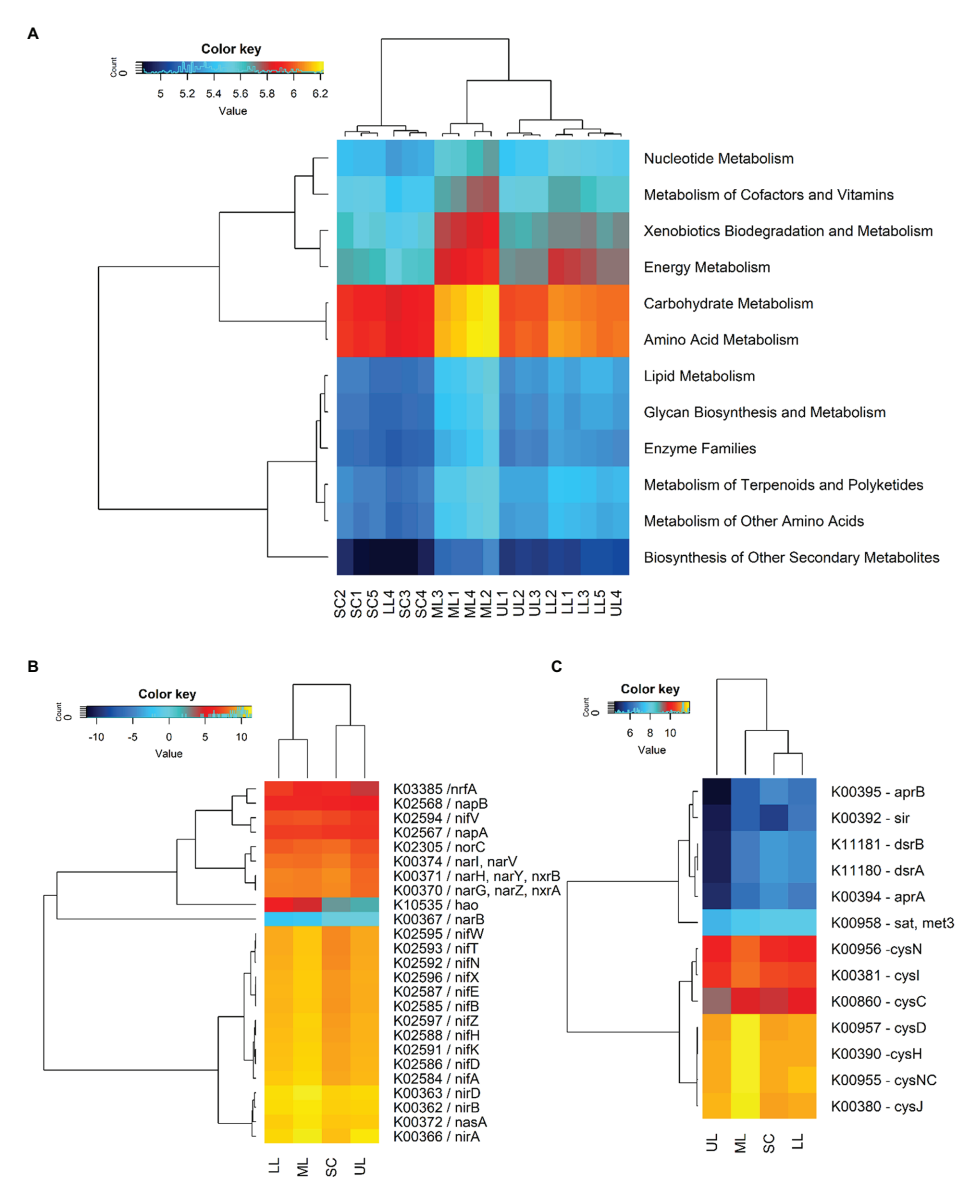
A heatmap showing the hierarchical clustering of the predicted KEGG Orthologs gene copy number (log2 transformed). **(A)** Clustering of metabolism pathways (level 2) of bacterial community across all samples: SC1-5, sugarcane farm; ML1-4, forest_mid; LL1-5, forest_lower; and UL, forest_upper. **(B)** N-metabolism genes. **(C)** S-metabolism genes. The dendrogram shows complete-linkage agglomerative clustering based on a Euclidean distance.

The predicted functions of the observed soil bacteria communities were determined by using PICRUSt2 analysis. The NSTI values for forest soil samples were in the range of 0.131–0.156, and 0.112–0.144 for the sugarcane farm soil samples, which were close to those reported for soil samples (Langille et al., 2013). A total of 5,418 KEGG Orthology (KOs) groups were obtained, of which 4,991 (92.1%) were assigned to six categories of biological metabolic pathways (primary functional level): metabolism, genetic information processing, environmental information processing, cellular processes, organismal systems, and human diseases. However, the study focused on predicted functional categories related to microbial-mediated metabolism (Figure 6A). Among the predicted metabolism subfunctions, amino acid metabolism, carbohydrate metabolism, energy metabolism, xenobiotics biodegradation, metabolism of cofactors and nucleotide metabolism ranked highest. The cluster analysis of gene copy number showed that the distances between forest samples were close, while sugarcane soils were located far from other samples, which was consistent with hierarchical clustering based on Bray-Curtis similarity coefficients of bacterial community compositions. The predicted gene copy number of amino acid metabolism, carbohydrate metabolism, energy metabolism, xenobiotics biodegradation and metabolism, metabolism of cofactors and nucleotide metabolism decreased in the assayed soil samples in the following order: forest_mid (ML) > forest_lower (LL) > forest_upper (UL) > SC.

The study also predicted the relative abundance of some key C, N, and S-cycling-related functional genes in different samples of coastal scarp forest topography types and sugarcane farms. Comparatively, genes related to C fixation in photosynthetic organisms (10) and metabolism (125) were significantly higher [*p*(FDR) < 0.05] in forest samples compared to sugarcane farm soils, with the predicted gene copy number decreasing in the following order: forest_mid > forest_lower > forest_upper > sugarcane farm. The key C fixation genes predicted included *maeB* (K00029, malate dehydrogenase), *GPT*, *ALT* (K00814, alanine transaminase), *pgK* (K00927, phosphoglycerate kinase), *ppdK* (K01006, pyruvate orthophosphate kinase), *pck*A [K01610, phosphoenolpyruvate carboxykinase (ATP)], *ALDO* (K01623, fructose-bisphosphate aldolase), *fbp* (K03841, fructose-1,6-bisphosphatase I), *fbp3* (K04041, fructose-1,6-bisphosphatase III), *GAPA* (K05298, glyceraldehyde-3-phosphate dehydrogenase), and *SHPK* (K11214, sedoheptulokinase; Supplementary Table 4). The relative abundance of some key N-cycling genes, primarily those involving N fixation (*nifA*, *nifD*, *nifK*, *nifH*, *nifZ*, *nifB*, *nifE*, *nifX*, *nifN*, *nifT*, *nifV*, and *nifW*), assimilatory and dissimilatory N reduction (*nasA*, *nasA*, *narB*, *nirB*, *nirA*, *narI*, and *nrfA*), nitrification (*hao*), and denitrification (*narG*, *narZ*, *nxrA*, and *norC*) were also detected (Figure 6B). Overall, forest samples were characterized by significantly higher (*p* < 0.05) levels of N fixing genes (*nif* genes), whereas sugarcane farm soils had higher nitrate reductases (K00371 *narH*, K00370 *narG*, and K00374 *narl*) and nitrite oxide reductase (K02305, *norC*). PICRUSt2 analysis also predicted the relative abundance of S-metabolism genes, closely related to assimilatory sulfite reductases (*cysJ*, *cysNC*, *cysH*, *cysD*, *cysC*, *cysI*, and *cysN*), adenylylsulfate reductase (*aprB* and *aprA*), dissimilatory sulfite reductase (*dsrB* and *dsrA*), and adenylyltransferase (*sat*, *met3*; Figure 6C). Similar to C and N cycling genes, higher gene copy number was predicted for forest_mid samples compared to other samples, with assimilatory sulfite reductases (*cysJ*, *cysNC*, *cysH*, *cysD*, *cysC*, *cysI*, and *cysN*) showing higher relative abundance. However, sugarcane farm soil samples clustered together with forest_mid and lower samples. Among all samples, forest_upper exhibited very low gene copy numbers for dissimilatory adenylylsulfate reductases (*aprB*, K00395; *aprA,* K00394), assimilatory sulfite reductase *sir* (K00392), and dissimilatory sulfite reductases (*dsrB*, K11181; *dsrA*, K11180).

## Discussion

In general, pristine forest biomes have specific features that differentiate them, and may provide unique perspective for the study of belowground-associated microbiota. As previously reported by Mucina et al. (2018), coastal scarp forests can be grouped into three forest subtypes dependent on the geographic location and gradients essentially associated to the local geomorphology. This study evaluated the three forest subtypes, differentiated by topography (gradients) and tree canopy coverage, to establish the relationships of the specific forest topography types, soil physicochemical characteristics, and the microbial community structure in a highly endemic pristine coastal scarp forest from Oribi Gorge, South Africa. Results showed that diversity of bulk soil bacterial community were clearly differentiated by the forest topography type (Table 2), which were represented by soil physical and chemical properties, of which the steeper forest_upper soils showed the most distinct characteristics in comparison with the other forest topography types (Table 1). Further, the subset of bacterial taxa distribution and functional differences in the communities were specific to each forest topography types, which were related to different phylum distributions (Figures 4–6). Overall, the adjacent sugarcane farm soils also had distinct bacterial diversities and community structures and functional differences from those of forest soils (Figures 2, 4–6).

### Forest Topography Type and Land Use Patterns Affects Soil Bacterial Community Structures

Assessment of overall bacterial community composition and diversity in forest soils revealed differences in the complexity of soil microbial communities across the three forest topography types. The alpha diversity indices (Chao1 and Simpson diversity) of soil microbial community were significantly higher (*p* < 0.05) in the less steep forest types (mid and lower) than in steeper forest type (upper; Table 2), indicating distinguishable community structure of the steeper forest soil. Further, the bacterial community structures were differentiated by the forest topography types, which were represented by differing plant density and soil physicochemical properties, as observed by PCA and NMDS analysis (Figure 3). Further analysis using RDA variation partitioning determined the relative contributions of forest topography type and soil properties on forest soil microbial community structure variation (Figure 3D). Unique contributions showed forest topography type and soil parameters (pH, SOM, and N) accounted for 7.3 and 14.9% variation in microbial community, respectively.

Consistent with findings of this study Bryant et al. (2008) and Wang et al. (2015) reported that bacterial taxon richness and phylogenetic diversity decreases monotonically with plant density from the lowest to highest elevations on the mountainsides. On the other hand, Singh et al. (2012) reported a hump-backed trend in bacterial diversity with elevation (0–3,500 m) on Mount Fuji, Japan, with the diversity not parallel to woody plant or herbaceous plant diversity. The study concluded that at mid elevations, less competition, and greater bacterial species diversity was higher due to “lottery” recruitment as a result of extreme temperature fluctuations, stronger UV, lack of nutrients, and more frequent disturbance of the loose substrate of these slopes. In contrast, the physiological challenges at the highest elevations are so extreme selecting for fewer bacterial species capable of surviving. In this study, the altitudinal differences were very small (<250 m asl), thus the contribution of the aforementioned factors on soil bacterial diversity perturbations could be minimal. Congruent to our results, however, Shen et al. (2015) also reported that distinct soil bacterial communities structure occurred along a small scale elevation gradient in Changbai Alpine tundra, China, dependent on the spatial heterogeneity of soil C and N contents.

Generally, forest topography type and soil parameters are interlinked. Local geomorphology is generally critical to the formation processes and spatial heterogeneity of forest topsoils, that may drive both microbial and plant diversity dynamics. Higher plant density and its associated forest canopy in the less steep forest topography can enhance soil organic C and N dynamics by increasing SOM input and decreasing the decomposition rate (Nadal-Romero et al., 2016; Xue et al., 2017), shaping the resource availability to the soil community. In this study, the less steep forest topography types were characterized by higher soil C/N ratio and SOM that correlated to the observed higher bacterial richness and diversity in the forest_lower and forest_mid soil samples (Table 2). In contrast, the steeper forest_upper (UL) are unique environment characterized by low plant density and stony and sandy acidic soils (pH < 5.0), generally low in nutrient reserves (low SOM and N) and cation exchange capacity (Mucina et al., 2018). The results of the study show that the nutrient-rich soils of less steep forest topography possess bacterial community structures, which are distinct from those in comparatively nutrient-reduced soils in steeper forest_upper (UL). The contribution of pH as a better predictor of soil bacterial elevational distribution as well as the indirect effect of vegetation types through altering soil C and N status has also previously been reported (Shen et al., 2013). In this study, the clustering of forest topography type bacterial diversity was significant and negatively correlated with acidic pH. Several studies have reported that pH is one of the most important in environmental factors in shaping the biogeographical patterns of microbial diversity in forest ecosystems (Lauber et al., 2009; Shen et al., 2013; Cho et al., 2016; Amoo and Babalola, 2019). Hence, it can be concluded that ecological niche-based environmental filtering processes related to soil C and N contents including pH might play a dominant role in structuring bacterial communities along the different coastal scarp forest topographies.

Coastal scarp forest are small and highly fragmented biomes restricted to the river gorges draining the large sugarcane plantations. Land use intensification has resulted in large forested lands conversion to farm lands. In this study, we hypothesized that land use change would significantly alter the soil bacterial community composition, with sugarcane farm soil harboring a distinct soil bacterial community driven by changes in soil chemistry. Consistent with this hypothesis, the adjacent sugarcane farm soils had distinct overall bacterial diversities and community structures from those of forest soils (Figures 2, 4, 5), mainly driven by soil pH, SOM, and N. Like the forest_upper, the sugarcane farm was characterized by significantly (*p* < 0.05) highly acidic soils (pH < 4.0), high N and low C/N ratio. In this study, the clustering of sugarcane farm soil bacterial diversity was positively correlated with pH. Previous studies have showed that soils under cane are more compacted, more acid, contain less organic matter and are lower in cation exchange capacity and exchangeable cations, that reflects soil degradation caused by intensive cultivation, leading to lower bacterial richness and diversity (Graham et al., 2002; Cherubin et al., 2015, 2016; Franco et al., 2015). The degradation of soils include soil compaction and structural breakdown occurring during harvest and cultivation operations, losses of organic matter due to burning of crop residues and acidification of soils due to large applications of nitrogen fertilizers (Cherubin et al., 2016), that may have an effect on the microbial diversity patterns.

### Distribution of Bacterial Taxa Across and Specific to Forest Topography Types

In terms of bacterial composition, our results demonstrated that scarp forest soils were dominated by seven bacterial phyla (*Proteobacteria*, *Actinobacteria*, *Actinobacteria*, *Planctomycetes*, *Firmicutes*, *Chloroflexi*, and *Bacteriodetes*) at higher taxa level. The relative average abundance of the top two major bacterial groups *Proteobacteria* (total 41.7%), *Acidobacteria* (12.8%), and *Planctomycetes* (10.3%) were comparable with results from other tropical (Miyashita, 2015; Tripathi et al., 2016) and temperate forests (Miyashita, 2015; Wei et al., 2018). However, the scarp forest soil bacterial diversity differed from other forest ecosystems with higher relative abundance of the phylum *Actinobacteria* and *Firmicutes* compared to phylum *Acidobacteria*. Further, subtle differences in bacterial community composition of forest topography types were discernible (Figures 3, 4; Supplementary Figure 2), showing dependence on pH and nutrient availability (SOM). Copiotrophic taxa, such as *α-proteobacteria*, *β-proteobacteria*, and *Actinobacteria* (Ho et al., 2017), are adapted to nutrient-rich environments, as found in the soils of forest_mid and forest_lower topographies (Table 1; Figure 2). In contrast, members *Acidobacteria*, *δ-proteobacteria*, and *Planctomycetes* are recognized as oligotrophs, which are adapted to nutrient-limiting conditions (Aires et al., 2016; Lladó et al., 2018; Dukunde et al., 2019). In this study, the nutrient-limited acidic soils of forest_upper favored actinobacterial and planctomycetous groups. Consistent with current findings, Dukunde et al. (2019) also reported higher abundance of members of *α-* and *β-proteobacteria* and *Actinobacteria* in nutrient-rich lime stand temperate deciduous forest soil, while abundance of acidobacterial groups were enriched in nutrient-poor and acidic beech stand temperate deciduous forest soils.

Proteobacterial genera were evenly distributed across all forest sites with slight differences between less steep and steeper forests topography, at total community level (Figure 4). The *Rhizobiales* (genera *Variibacter*, *Bradyrhizibium*, and unclassified *Rhizobiales*) and *Rhodospirallales* (unclassified *Rhodospirillum* DA111) within the *α-proteobacteria* were more abundant in forest mid and lower topographies, whereas *β-proteobacteria* order *Burkholderiales* was enriched in forest upper soils. *Rhizobiales* have previously broadly linked with nitrogen fixation, plant pathogenicity, and organic matter decomposition, and play important role in nutrient recycling in forest ecosystems (Fierer and Jackson, 2006; Lladó et al., 2017; Dukunde et al., 2019). Likewise, *β-Proteobacteria* is one of the most important groups in soils with relevance in N circulation, with orders *Burkholderiales* and *Nitrosomonadales* being part of the nitrogen-fixing bacterial community in forests soils, generally participating in symbiotic relationships with plants. Interestingly, the most differentially abundant of taxa in forest_upper was *Burkholderia-Paraburkholderia*, a bacterial genus that is frequently found in low pH environments contributing to multiple ecological processes such plant litter decomposition (both cellulose and lignin) in forest and agricultural soils (Berkelmann et al., 2018; Nicolitch et al., 2019) and mineral weathering (Colin et al., 2017; Samuels et al., 2020). Consistent with these results, correlation-based indicator species analysis (Cáceres and Legendre, 2009) also revealed that *Burkholderia-Paraburkholderia* was one of the unique genera distinguishing steeper topography from other forest sites (Figure 5; Supplementary Table 3). Other members of order *Burkholderiales* associated with forest upper included genus *Delftia* and *Massilia*. Whereas *Delftia* are plant growth-promoting rhizobacterium characterized by the ability to transform or degrade a wide variety of organic and inorganic compounds including metallic ions (Braña et al., 2016), members of genus *Massilia* are important degraders of complex polysaccharides in soils (Wieczorek et al., 2019). Collectively, the differential abundance of these taxa suggests the key role they play in nutrient cycling and mineral weathering, mainly in the highly leached sandy/stony and nutrient-poor acidic soils characteristic of the steeper forest upper ecosystems.

Comparatively, Actinobacterial taxa was dominant in the sugarcane soil samples (accounting total 46.4%) compared to forest soils (18.6%). Similar to our finding, Navarrete et al. (2015) reported higher abundance of phylum *Actinobacteria* in 57 soil metagenomic sets of sugarcane farms practicing straw retention. Other differentially significant phyla between forest and sugarcane farm samples were *Planctomycetes*, *Firmicutes*, *Chloroflexi*, *Nitrospirae*, and *Armatimonadetes*, that have been reported in other studies of forest and farmland soil ecosystems (Lauber et al., 2009; Shen et al., 2013; Cho et al., 2016; Amoo and Babalola, 2019). Indicator species analysis and bipartite association networks also provided insight into bacterial taxa, mainly, 12 OTUs associated to phylum *Actinobacteria* (mainly members of the order *Frankiales*, *Acidimicrobiales*, and *Solirubrobacterales*) that potentially drive the observed community structures in the sugarcane farm soil (Figure 5). Genus *Acidothermus* (order *Frankiales*) and uncultured *Solirubrobacterales* YNFP111 were the most abundant actinobacterial indicator taxa in sugarcane farm soil.

Under sugarcane farming, the soil C is greatly reduced that may greatly influence the bacterial diversity and composition. Depletion of easily available C pools, typical of the sugarcane farm, has been associated with increased dominance of phylum *Actinobacteria* (Barret et al., 2011). Members of *Frankiales*, *Acidimicrobiales*, and *Solirubrobacterales* orders have been reported to have many representative taxa that produce a range of extracellular hydrolytic enzymes which can degrade plant polymers, including lignin, cellulose, and other organic compounds (Eisenlord and Zak, 2010). Other unique taxa driving community structure in sugarcane farm soils include the potentially cellulolytic genera in the phylum *Firmicutes* (*Paenibacillus* and *Bacillus*) involved in organic matter decomposition and *α-Proteobacteria* genus *Rhizomicrobium* and unclassified *Rhodospirillales* that may participate in organic matter decomposition nitrogen fixation (Carvalho et al., 2010; Dukunde et al., 2019). Members of genus *Acidothermus* exhibited higher abundance in the more acidic sugarcane farm soils compared to the less acidic forest soil. In contrast, the relative abundance and prevalence of copiotrophic *α-Proteobacteria* taxa (genus Rhizomicrobium and unclassified *Rhodospirillales*) reduced in both nutrient poor sugarcane farm and forest soils (Forest_upper) compared to other sites. Similar to these findings, enrichment of genus *Acidothermus* and other actinobacterial taxa such as *Acidimicrobiales* in farmland soils have been reported to be negatively correlated to soil pH (Wang et al., 2019). Liu et al. (2018) also reported the abundance of *Proteobacteria* in the soils of Loess plateau in China was dependent on the nutrient content and pH. Thus, these bacterial groups collectively contribute significantly to energy metabolism process, specifically C and N cycling, but the contribution of nutrient availability and soil pH as drivers of unique microbial community observed within the sugarcane farm soil is important.

### Bacterial Functional Profiles Across Forest topography

To delineate the potentially active metabolic processes important in biogeochemical cycles in the forest soils, several studies have used 16S rDNA-based communities prediction algorithms (Cardenas et al., 2018; Dukunde et al., 2019). In this study, we observed several predicted gene functional profiles related to carbon fixation and metabolism (Figure 6). Consistent with our findings, several studies have reported higher abundance of genes related to carbon metabolism, indicating the key role played by bacterial community in the C cycling within nutrient-rich (high SOC) forest soils (Siles and Margesin, 2017; Dukunde et al., 2019). We also found higher abundance of potential cellulolytic genera (Ulrich et al., 2008; Schellenberger et al., 2010; Štursová et al., 2012) such as α-proteobacterial genera (*Variibacter* and *Bradyrhizobium*, in the forest mid and lower topography), *β-proteobacteria* (*Burkholderia-Paraburkholderia*, in the forest upper soils), and *Actinobacteria* and *Firmicutes* genera (uncultured *Solirubrobacterales* YNFP111, unclassified *Actinobacteria*, *Bacillus*, and *Paenibacillus*, in the sugar cane soils). Further, gene functions related to N cycling main processes (fixation, denitrification, and nitrate reduction) such as *nir*, *nif*, *hao*, and *nar* were also observed. Comparatively, gene function for N metabolism was significantly more abundant in the forest_mid topography soils, however, sugarcane farm soils exhibited higher abundance of nitrate reductase genes (K00371 *narH*, K00370 *narG*, and K00374 *narl*; Figure 6). In sugarcane farming, high rates of inorganic fertilizer application to boost soil N has been associated with the perturbation of both soil physicochemical parameters such pH and microbiota diversity selecting specific groups such as *Nitrospiraceae*, members of *β-proteobacteria* and *γ-proteobacteria* in addition to *Actinobacteria* (Navarrete et al., 2015). In this study, sugarcane farm soils were also associated with higher abundance of members of *β-proteobacteria* and *γ-proteobacteria* in addition to *Actinobacteria* that may play a significant role in N cycling. While *γ-proteobacteria* have been reported to prefer soil environments with low pH and high C/N ratios, which promote efficient denitrification (Van Den Heuvel et al., 2010), *Nitrospiraceae* is highly physiologically diverse and includes chemolithoautotrophic aerobic nitrite-oxidizing bacteria that can use organic and inorganic N and straw mineralization (Navarrete et al., 2015; Ushiki et al., 2018). Overall, it should be noted that the diversity of N and C cycling bacterial community members occurs across several taxonomic groups, implying that bacterial metabolism of both C and nitrogen is closely interlinked (Dukunde et al., 2019). Thus, any perturbations in the soil C and N level within the forest ecosystem will affect microbiome-driven C and N cycling processes with ultimate impact on the overall ecosystem functioning.

## Conclusion

In conclusion, our study indicates that the microbial community structure and diversity of the soil in the coastal scarp forests are highly dependent on the local topography. This supported our first hypothesis that the local topographical/geomorphological gradients [as represented by the mean elevation and gradient (slope degree, aspect, and position)] affects both soil characteristics, forest vegetation, canopy coverage that indirectly drives the bacterial community structure of forest bulk soils. The highly leached sandy/stony soils low in organic nutrients (C and N) of the steeper gradients (forest_upper) and its associated low plant densities was correlated to decrease in bacterial taxonomic abundance. Overall, changes in bacterial community diversity in forest soils were associated with variation in pH, supporting diverse bacterial groups. In support of the second hypothesis, highly acidity (low pH) and availability of soil nutrients (TN and SOM) greatly influenced bacterial community structure in the sugarcane farm soils, selecting for high abundance of members of *Actinobacteria* and to some extent enrichment of *β-proteobacteria* and *γ-proteobacteria*. However, it should be noted this study focused on a small sampling area (~1,600 ha) of the coastal scarp forest ecosystems. Owing to the highly fragmented nature of the scarp forest ecosystem, several forest ecotypes dependent on local geomorphological formation but with varying plant endemism have been reported (Mucina et al., 2018), thus, the results of the current study do not necessarily give a complete reflection of the coastal scarp forest microbial diversity. Further, it is necessary to acknowledge the limitations of inferring biogeochemical function from *in silico* predictions such as PICRUSt algorithm (Douglas et al., 2018). Nevertheless, the current study gives a snapshot of the bacterial community diversity as affected by local topography and the potential effect of land use intensification on soil microbial community dynamics in the ecological significant coastal scarp forest ecosystem. Forests are generally dynamic on a broad temporal scale with processes ranging from short-term events over seasonal ecosystem dynamics affecting the microbial diversity (both fungi and bacteria) as shaped by a combination of many drivers such as local geomorphological/soil factors, vegetation composition, climatic factors, and anthropogenic influences (Lladó et al., 2018). Thus, further research is needed to explore the relationships between a large repertoire of spatial, temporal, and biogeochemical factors, and the microbial community structure (including other major microbial groups, such as fungi and archaea), which will help understand of the complex “ecosystem microbiome” and its functioning unique to coastal scarp forest biome.

## Data Availability Statement

The datasets presented in this study can be found in online repositories. The names of the repository/repositories and accession number(s) can be found in the article/Supplementary Material.

## Author Contributions

HO designed the study, analyzed the data, and wrote the first draft of the paper. HO and RS carried out the experiments.MT supported the research. All authors contributed to the article and approved the submitted version.

### Conflict of Interest

The authors declare that the research was conducted in the absence of any commercial or financial relationships that could be construed as a potential conflict of interest.
