# Supplementary material for: Local Geomorphological Gradients and Land Use Patterns Play Key Role on the Soil Bacterial Community Diversity and Dynamics in the Highly Endemic Indigenous Afrotemperate Coastal Scarp Forest Biome
Source: Front Microbiol. 2021 Feb 24;12:592725. doi: 10.3389/fmicb.2021.592725 (PMC7943610; doi:10.3389/fmicb.2021.592725)
Supplement: Supplementary file 1 [file Data_Sheet_1.docx]

***Supplementary Tables***

**Supplementary Table 1.** Evaluation of variation between bacterial communities associated with different bulk soil samples by analysis of similarities (ANOSIM) and permutational multivariate analysis of variance (PERMANOVA) (Anderson and Walsh, 2013)

**Supplementary Table 2.** Environmental variables analyzed to explain possible dissimilarities in bacterial community composition among different forest topography and sugarcane farm soils using the envfit function of the vegan package in R. Permutation: free. Number of permutations: 999.

**Supplementary Table 3.** Bacterial genera significantly associated with distinct forest and forest landscape representing a broad taxonomic spectrum. The generalized indicator value (IndVal.g) and corresponding p-values used to assess the predictive value of a taxon for each forest topography category as implemented in *indicspecies* in R *(de Cáceres et al., 2010)* is given.

**Supplementary Table 4.** Predicted carbon cycle related pathways in the forest and sugarcane farm soil samples.

**Supplementary Table 1.** Evaluation of variation between bacterial communities associated with different bulk soil samples by analysis of similarities (ANOSIM) and permutational multivariate analysis of variance (PERMANOVA) (Anderson and Walsh, 2013)

|  |  | **ANOSIM**  **Bray-Curtis** | |  | **PERMANOVA**  **Adonis** | |
| --- | --- | --- | --- | --- | --- | --- |
|  |  | **R** | ***p*** |  | ***F*** | ***p*** |
| **Class** | Habitat | 0.6322 | 0.004** |  | 21.42 | 0.0003*** |
|  | Elevation | 0.6426 | 0.001** |  | 6.49 | 0.0001*** |
|  | LL *vs* UL | 0.218 | 0.112 |  | 1.92 | 0.1126 |
|  | LL *vs* ML | 0.289 | 0.031* |  | 2.04 | 0.0231* |
|  | LL *vs* SC | 0.932 | 0.008** |  | 11.73 | 0.0082** |
|  | UL *vs* ML | 0.490 | 0.028* |  | 3.60 | 0.0281* |
|  | UL *vs* SC | 0.881 | 0.009** |  | 11.16 | 0.0090** |
|  | ML *vs* SC | 0.981 | 0.008** |  | 12.23 | 0.0072** |
| **Genus** | Habitat | 0.493 | 0.0003*** |  | 3.72 | 0.0027** |
|  | Elevation | 0.439 | 0.0011** |  | 4.51 | 0.0003*** |
|  | LL *vs* UL | 0.163 | 0.142 |  | 2.38 | 0.087 |
|  | LL *vs* ML | 0.100 | 0.231 |  | 1.17 | 0.292 |
|  | LL *vs* SC | 0.536 | 0.009** |  | 4.99 | 0.010* |
|  | UL *vs* ML | 0.521 | 0.031* |  | 3.72 | 0.029* |
|  | UL *vs* SC | 0.888 | 0.008** |  | 6.41 | 0.008* |
|  | ML *vs* SC | 0.769 | 0.009** |  | 7.29 | 0.007* |

**Supplementary Table 2.** Environmental variables analyzed to explain possible dissimilarities in bacterial community composition among different forest topography and sugarcane farm soils using the envfit function of the vegan package in R. Permutation: free. Number of permutations: 999.

| **Parameter** | **NMDS1** | **NMDS2** | **R^2^** | **p-value** | **Significance** |
| --- | --- | --- | --- | --- | --- |
| **pH** | **-0.6501** | **-0.1234** | **0.6703** | **0.001** | ****** |
| SM | 0.0493 | 0.0127 | 0.0133 | 0.890 | - |
| BD | 0.0145 | -0.1982 | 0.3411 | 0.455 | - |
| **TN** | **0.3567** | **0.2431** | **0.1130** | **0.039** | ***** |
| **TC** | **0.4343** | **0.5167** | **0.6112** | **0.049** | ***** |
| **SOM** | **0.6713** | **0.2567** | **0.7311** | **0.007** | ****** |
| Ca | -0.1386 | -0.1807 | 0.2013 | 0.098 | - |
| Al | 0.1835 | 0.2277 | 0.1130 | 0.092 | - |
| Fe | -0.0763 | 0.0607 | 0.2411 | 0.996 | - |
| K | 0.2619 | 0.2065 | 0.2931 | 0.231 | - |
| Mg | 0.0400 | 0.1027 | 0.0123 | 0.881 | - |

Significant codes: 0.001, ‘***’; 0.01, ‘**’; 0.05, ‘*’

| **Supplementary Table 3.** Bacterial genera significantly associated with distinct forest and forest landscape representing a broad taxonomic spectrum. The generalized indicator value (IndVal.g) and corresponding p-values used to assess the predictive value of a taxon for each forest topography category as implemented in *indicspecies* in R *(de Cáceres et al., 2010)* is given. | | | | | | | | | | | | |
| --- | --- | --- | --- | --- | --- | --- | --- | --- | --- | --- | --- | --- |
| **Genera** | **IndVal** | | | | **p-value** |  | **Genera** | **IndVal** | | | | **p-value** |
|  | **LL** | **ML** | **UL** | **SC** |  |  |  | **LL** | **ML** | **UL** | **SC** |  |
| Saccharopolyspora | 0.920 | - | - | - | 0.001 |  | Alicyclobacillus | - | - | 0.509 | - | 0.009 |
| Alphaproteobacteria_uc | 0.827 | - | - | - | 0.003 |  | Ramlibacter | - | - | 0.501 | - | 0.017 |
| Isosphaera | 0.788 | - | - | - | 0.002 |  | Erythrobacteraceae_unclassified | - | - | 0.490 | - | 0.035 |
| uncultured_Planctomycetaceae | 0.779 | - | - | - | 0.002 |  | Micrococcales_unclassified | - | - | 0.486 | - | 0.045 |
| Candidatus_Nostocoida | 0.732 | - | - | - | 0.005 |  | Ralstonia | - | - | 0.472 | - | 0.031 |
| Planctomycetaceae_uc | 0.707 | - | - | - | 0.009 |  | Caenimonas | - | - | 0.470 | - | 0.041 |
| Lachnospiraceae_uc | 0.685 | - | - | - | 0.012 |  | FBP_ge | - | - | 0.452 | - | 0.028 |
| Subgroup_13_ge | 0.654 | - | - | - | 0.018 |  | Actinobacteria_uc | - | - | - | 0.927 | 0.001 |
| Pelosinus | 0.653 | - | - | - | 0.018 |  | Acidothermus | - | - | - | 0.911 | 0.001 |
| Subgroup_6_uc |  | 0.680 | - | - | 0.018 |  | Solirubrobacterales_uc | - | - | - | 0.874 | 0.001 |
| Intrasporangium | - | 0.679 | - | - | 0.034 |  | Nocardiaceae_uc | - | - | - | 0.818 | 0.002 |
| Lineage_IIb_ge | - | 0.679 | - | - | 0.034 |  | YNPFFP1_ge | - | - | - | 0.798 | 0.002 |
| Rhodospirillaceae_uc | - | 0.667 | - | - | 0.018 |  | Turicibacter | - | - | - | 0.777 | 0.004 |
| Sphingomonas | - | 0.661 | - | - | 0.028 |  | GAL15_ge | - | - | - | 0.760 | 0.005 |
| Acidimicrobiaceae_uc | - | 0.609 | - | - | 0.045 |  | Rhodospirillales_uc | - | - | - | 0.725 | 0.007 |
| JG34-KF-161_ge | - | 0.609 | - | - | 0.049 |  | Rhizomicrobium | - | - | - | 0.720 | 0.008 |
| Burkholderia-Paraburkholderia | - | - | 0.848 | - | 0.002 |  | Streptomycetaceae_uc | - | - | - | 0.718 | 0.011 |
| Neisseriaceae_uc | - | - | 0.788 | - | 0.001 |  | Mycobacterium | - | - | - | 0.713 | 0.010 |
| MNG7_ge | - | - | 0.782 | - | 0.010 |  | Actinoallomurus | - | - | - | 0.709 | 0.008 |
| Arenimonas | - | - | 0.779 | - | 0.003 |  | Actinospica | - | - | - | 0.702 | 0.015 |
| Betaproteobacteria_uc | - | - | 0.779 | - | 0.003 |  | Nocardia | - | - | - | 0.690 | 0.009 |
| Aquicella | - | - | 0.749 | - | 0.002 |  | Corynebacteriales_u | - | - | - | 0.667 | 0.020 |
| Candidatus_Alysiosphaera | - | - | 0.723 | - | 0.015 |  | TK10_ge | - | - | - | 0.649 | 0.021 |
| Coxiellaceae_uc | - | - | 0.723 | - | 0.005 |  | uncultured_Family_XVII | - | - | - | 0.616 | 0.014 |
| uncultured_Alcaligenaceae | - | - | 0.719 | - | 0.008 |  | Pseudoclavibacter | - | - | - | 0.586 | 0.044 |
| Armatimonadales_ge | - | - | 0.692 | - | 0.012 |  | Fontibacillus | 0.577 | - | 0.577 | - | 0.050 |
| Gammaproteobacteria_uc | - | - | 0.688 | - | 0.017 |  | Acetobacteraceae_uc | 0.811 | - | - | 0.811 | 0.001 |
| Bacillales_uc | - | - | 0.685 | - | 0.020 |  | Acidobacterium | 0.786 | - | - | 0.786 | 0.002 |
| Comamonadaceae_uc | - | - | 0.678 | - | 0.009 |  | uncultured_Actinobacteria_ge | 0.762 | - | - | 0.762 | 0.001 |
| Sphingobacteriales_uc | - | - | 0.676 | - | 0.014 |  | Acidimicrobiales_uc | 0.716 | - | - | 0.716 | 0.011 |
| Bdellovibrio | - | - | 0.671 | - | 0.017 |  | Roseiarcus | 0.714 | - | - | 0.714 | 0.011 |
| Flavitalea | - | - | 0.670 | - | 0.033 |  | Jatrophihabitans | 0.706 | - | - | 0.706 | 0.010 |
| uncultured_Nitrosomonadaceae | - | - | 0.667 | - | 0.019 |  | Solibacteraceae_(Subgroup_3) | 0.689 | - | - | 0.689 | 0.013 |
| Delftia | - | - | 0.666 | - | 0.024 |  | Candidatus_Koribacter | 0.667 | - | - | 0.667 | 0.021 |
| Duganella | - | - | 0.662 | - | 0.015 |  | Bryobacter | 0.658 | - | - | 0.658 | 0.026 |
| Archangiaceae_unclassified | - | - | 0.661 | - | 0.012 |  | Edaphobacter | 0.653 | - | - | 0.653 | 0.024 |
| 0319-6G20_ge | - | - | 0.659 | - | 0.025 |  | DA111_ge | 0.652 | - | - | 0.652 | 0.024 |
| Bacilli_unclassified | - | - | 0.652 | - | 0.010 |  | Variibacter | 0.626 | - | - | 0.626 | 0.044 |
| Opitutus | - | - | 0.639 | - | 0.029 |  | Granulicella | 0.615 | - | - | 0.615 | 0.049 |
| Pseudoduganella | - | - | 0.590 | - | 0.013 |  | Schlesneria | - | 0.716 | 0.716 | - | 0.011 |
| uncultured_Fibrobacteraceae | - | - | 0.589 | - | 0.006 |  | mle1-27_ge | - | 0.692 | 0.692 | - | 0.012 |
| Terrimonas | - | - | 0.589 | - | 0.030 |  | Mesorhizobium | - | 0.688 | 0.688 | - | 0.016 |
| Paucimonas | - | - | 0.556 | - | 0.045 |  | Planctomyces | - | 0.632 | 0.632 | - | 0.032 |
| Ferruginibacter | - | - | 0.547 | - | 0.035 |  | Legionella | - | 0.630 | 0.630 | - | 0.036 |
| Pla4_lineage_ge | - | - | 0.547 | - | 0.024 |  | Bauldia | - | 0.618 | 0.618 | - | 0.044 |
| Massilia | - | - | 0.537 | - | 0.030 |  | Actinobacteria | - | 0.755 | - | 0.755 | 0.006 |
| Oxalobacteraceae_unclassified | - | - | 0.525 | - | 0.032 |  |  |  |  |  |  |  |

**Supplementary Table 4.** Predicted carbon cycle related pathways in the forest and sugarcane farm soil samples.

| **Pathway** | **Definition** | **Module** | **Orthology** | **p-value** | **p- (FDR)** | **forest soil** | **Sugarcane farm soil** |
| --- | --- | --- | --- | --- | --- | --- | --- |
| ko01200 | Carbon metabolism | M00020, M00021, M00356, M00346, M00309, M00344, M00345, M00374, M00010, M00011, M00012, M00002, M00004, M00005, M00006, M00001, M00375, M00377, M00007, M00008, M00009, M00013, M00373, M00376, M00167, M00170, M00171, M00172, M00740, M00741, M00173, M00620, M00633, M00532, M00563, M00567, M00579, M00580, M00174, M00168, M00307, M00308, M00165, M00166, M00169, M00357 | K00023, K00027, K00029, K00030, K00036, K00043, K00121, K00123, K00124, K00126, K00131, K00140, K00148, K00161, K00162, K00171, K00192, K00197, K00198, K00200, K00201, K00202, K00203, K00204, K00205, K00209, K00234, K00236, K00237, K00240, K00245, K00246, K00247, K00248, K00249, K00261, K00317, K00320, K00382, K00605, K00626, K00814, K00845, K00850, K00886, K00926, K00927, K01006, K01053, K01070, K01455, K01499, K01610, K01623, K01676, K01677, K01679, K01738, K01752, K01782, K01825, K01849, K01899, K01958, K01961, K01962, K01963, K01964, K01966, K02160, K02437, K03396, K03737, K03738, K03841, K04041, K05298, K05299, K05308, K05606, K07404, K07514, K07515, K07516, K08093, K08094, K08691, K10713, K10714, K10946, K11263, K11389, K11517, K11529, K13788, K13810, K13831, K13942, K14067, K14080, K14081, K14126, K14138, K14447, K14449, K14465, K14468, K14470, K14472, K15024, K15230, K15231, K15233, K15634, K15916, K17066, K17989, K18125, K18209, K18210, K18472, K18557, K18560, K18978, K19243 | 0.034 | 0.048 | 1.896 | 1.373 |
| ko00710 | Carbon fixation in photosynthetic organisms | M00374, M00375, M00377, M00376, M00167, M00170, M00171, M00172, M00173, M00620, M00579, M00168, M00165, M00166, M00169 | K00029, K00814, K00927, K01006, K01610, K01623, K03841, K04041, K05298, K11214 | 0.034 | 0.049 | 0.182 | 0.171 |
| ko00680 | Methane metabolism | M00358, M00356, M00346, M00344, M00345, M00378, M00422, M00563, M00608, M00567, M00174, M00357 | K00093, K00121, K00123, K00124, K00126, K00148, K00171, K00192, K00197, K00198, K00200, K00201, K00202, K00203, K00204, K00205, K00317, K00320, K00400, K00440, K00443, K00850, K01070, K01499, K01623, K03396, K03841, K04041, K05299, K07072, K07144, K08093, K08094, K08097, K08691, K09733, K10713, K10714, K10946, K11212, K11529, K11781, K12234, K13788, K13831, K13942, K14067, K14080, K14081, K14126, K14941, K15229, K15634, K16256, K16793, K17066, K18277, K22081, K22082, K22083, K22084, K22085, K22086, K22087, K22305 | 0.021 | 0.026 | 0.595 | 0.613 |
| ko00620 | Pyruvate metabolism | M00307 | K00016, K00027, K00029, K00049, K00090, K00101, K00138, K00156, K00158, K00161, K00162, K00171, K00245, K00246, K00247, K00382, K00626, K01006, K01026, K01572, K01573, K01610, K01676, K01677, K01679, K01905, K01946, K01958, K01961, K01962, K01963, K02160, K03737, K04072, K05523, K11263, K13788, K15024, K17741, K18472, K19266, K22212 | 0.009 | 0.010 | 0.896 | 0.918 |
| ko00190 | Oxidative phosphorylation | M00150, M00156, M00142, M00153, M00143, M00144, M00145, M00146, M00147, M00148, M00149, M00151, M00152, M00154, M00155, M00416, M00417, M00157, M00158, M00159, M00160 | K00234, K00236, K00237, K00240, K00245, K00246, K00247, K00332, K00333, K00334, K00337, K00339, K00340, K00341, K00342, K00343, K00404, K00405, K00406, K00407, K00410, K00411, K00413, K00424, K01541, K01544, K02117, K02118, K02119, K02120, K02121, K02122, K02123, K02124, K02152, K02258, K02259, K02261, K02277, K02298, K02299, K02300, K02827, K02828, K03881, K03883, K03886, K03887, K03888, K03889, K03890, K03891, K03941, K03954, K05573, K06019, K11352, K11725, K11726, K15408, K15862, K15986 | 0.004 | 0.004 | 1.026 | 0.993 |

**References**

Anderson, M. J., and Walsh, D. C. I. (2013). PERMANOVA, ANOSIM, and the Mantel test in the face of heterogeneous dispersions: what null hypothesis are you testing? *Ecol. Monogr.* 83, 557–574.
