# Supplementary material for: Local Geomorphological Gradients and Land Use Patterns Play Key Role on the Soil Bacterial Community Diversity and Dynamics in the Highly Endemic Indigenous Afrotemperate Coastal Scarp Forest Biome
Source: Front Microbiol. 2021 Feb 24;12:592725. doi: 10.3389/fmicb.2021.592725 (PMC7943610; doi:10.3389/fmicb.2021.592725)
Supplement: Supplementary file 2 [file Data_Sheet_2.pdf]

# *Supplementary Figures*

**Supplementary Figure 1.** Rarefaction curve and rank abundance plots

**Supplementary Figure 2.** Relative abundance of the top 15 classes. Heatmap was generated by ampvis2 package in R computing environment version 3.5.2.

**Supplementary Figure 3.** Venn diagram of unique and shared bacterial OTUs (at the 3% evolutionary distance) within the 3 topography types and between forest and sugarcane farm.

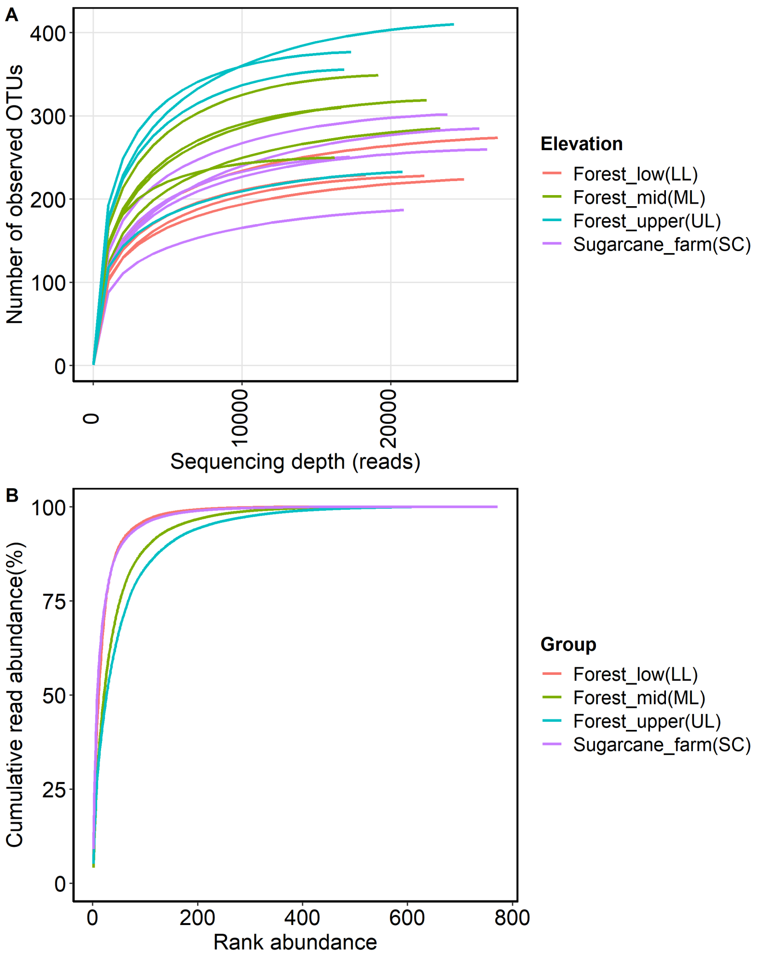


**Supplementary Figure 1.** Rarefaction curve and rank abundance plots

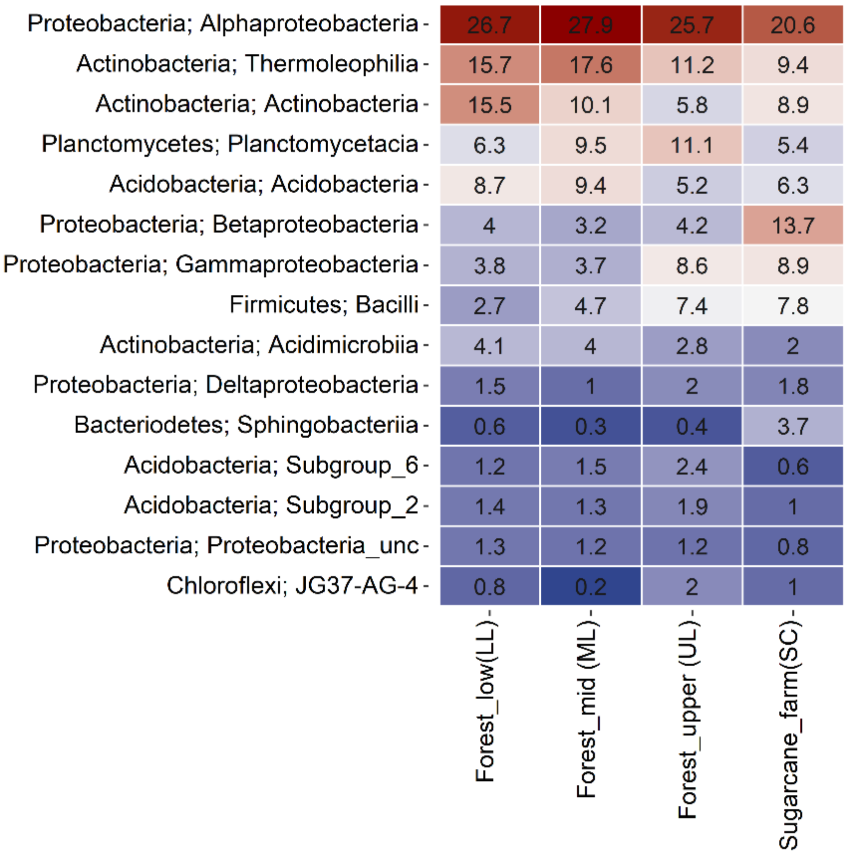


**Supplementary Figure 2.** Relative abundance of the top 15 classes. Heatmap was generated by *ampvis2* package in R computing environment version 3.5.2.

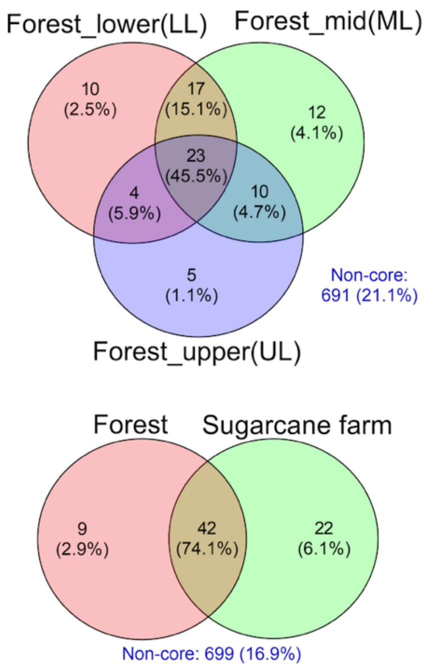


**Supplementary Figure 3.** Venn diagram of unique and shared bacterial OTUs (at the 3% evolutionary distance) within the 3 topography types and between forest and sugarcane farm.
